# Fluorescence-Based Mono- and Multimodal Imaging for In Vivo Tracking of Mesenchymal Stem Cells

**DOI:** 10.3390/biom13121787

**Published:** 2023-12-13

**Authors:** Wan Su Yun, Hanhee Cho, Seong Ik Jeon, Dong-Kwon Lim, Kwangmeyung Kim

**Affiliations:** 1 KU-KIST Graduate School of Converging Science and Technology, Korea University, Seoul 02841, Republic of Korea; wsyun91@gmail.com (W.S.Y.); dklim@korea.ac.kr (D.-K.L.); 2 Graduate School of Pharmaceutical Sciences, College of Pharmacy, Ewha Woman’s University, Seoul 03760, Republic of Korea; ricky@ewha.ac.kr (H.C.); jeonseongik@gmail.com (S.I.J.)

**Keywords:** stem cell tracking, fluorescence imaging, multimodal imaging, in vivo cell imaging

## Abstract

The advancement of stem cell therapy has offered transformative therapeutic outcomes for a wide array of diseases over the past decades. Consequently, stem cell tracking has become significant in revealing the mechanisms of action and ensuring safe and effective treatments. Fluorescence stands out as a promising choice for stem cell tracking due to its myriad advantages, including high resolution, real-time monitoring, and multi-fluorescence detection. Furthermore, combining fluorescence with other tracking modalities—such as bioluminescence imaging (BLI), positron emission tomography (PET), photoacoustic (PA), computed tomography (CT), and magnetic resonance (MR)—can address the limitations of single fluorescence detection. This review initially introduces stem cell tracking using fluorescence imaging, detailing various labeling strategies such as green fluorescence protein (GFP) tagging, fluorescence dye labeling, and nanoparticle uptake. Subsequently, we present several combinations of strategies for efficient and precise detection.

## 1. Introduction

The landscape of regenerative medicine has undergone a paradigm shift with the integration of stem cells, opening up previously uncharted territories in therapeutic solutions [1,2,3]. These undifferentiated cells hold an extraordinary ability to morph into specialized cells, paving the way for innovative treatments across a spectrum of diseases. This transformation has instilled hope in the realm of medical treatments, particularly for conditions that were once perceived as insurmountable challenges. For example, stem cells hold particular significance in addressing neurological afflictions such as Parkinson’s and Alzheimer’s disease [4,5]. Beyond the confines of the neural realm, these cells have shown immense potential for remedying cardiac complications and accelerating bone regeneration processes [6,7]. This broad applicability is a testament to their intrinsic capability to differentiate and renew themselves, making them a cornerstone in the field of regenerative medicine. The core essence of stem cell application pivots on their remarkable capacity to address a wide array of diseases, including those that have, until now, remained elusive to definitive treatments. Several groundbreaking studies and clinical trials have shed light on stem cells’ potential in not just regenerating damaged tissues but also in replacing cells that have lost their function due to disease or age [8,9,10]. Furthermore, the recent advances in targeted drug delivery have amplified the role of stem cells as vectors, enabling them to deliver therapeutic agents directly to specific disease sites [11,12,13].

Harnessing the transformative power of stem cells in regenerative medicine hinges crucially on the capacity to meticulously monitor their post-transplantation behavior and trajectory [14,15,16]. This is not just a matter of scientific curiosity; it is an essential prerequisite to ensure that these cellular therapies are both safe and efficacious [16,17,18,19,20,21]. In the absence of robust in vivo imaging and tracking systems, navigating the intricate pathways of stem cell therapy becomes akin to flying blind. Without these tracking mechanisms, it is challenging to know if the transplanted cells are going to the right tissues, proliferating at the right rate, or differentiating into the desired cell types [22]. Moreover, without real-time tracking, detecting unforeseen side effects or aberrant behaviors becomes a challenge, posing potential risks to patient safety. Beyond these considerations, in vivo, stem cell tracking provides invaluable insights for optimizing therapeutic protocols [23]. It can inform dosage determinations, shed light on the best routes of administration, and highlight the most effective stem cell types for particular applications. Such insights can be pivotal in refining treatments, ensuring that patients receive the maximum therapeutic benefit with minimal risks. Furthermore, real-time monitoring paints a dynamic picture of stem cell behavior [24,25,26,27]. Observing stem cell distribution, engraftment, and differentiation in a living organism offers a window into their interactions with the host environment. Such insights can provide clues about why certain therapies succeed while others falter, guiding the next steps in research and clinical applications.

Therefore, considering the importance of in vivo imaging and tracking of stem cells, as previously mentioned, several imaging strategies have been developed. Furthermore, in the expansive toolbox of in vivo stem cell tracking techniques, fluorescence imaging stands out as a luminescent choice. While it has heavyweight counterparts such as MRI, CT, and PET, fluorescence imaging offers its own unique advantages rooted in its fundamental principles. Fluorescence imaging operates based on the behavior of molecules known as fluorophores. When these molecules absorb photons of a certain wavelength, they enter an ‘excited’ state. In seeking stability, they return to their ‘ground’ state, releasing energy in the form of photons of light. Crucially, these emitted photons have a longer wavelength than the absorbed ones, producing a discernible color difference known as the ‘Stokes shift’. This characteristic is key for the detection and imaging of fluorophores. 

What makes fluorescence imaging especially attractive for stem cell tracking is its high spatial resolution, which allows for the intricate visualization of cellular and subcellular structures [28,29]. This clarity is pivotal in understanding the nuanced behaviors of stem cells post-transplantation. Furthermore, fluorescence imaging offers real-time monitoring capabilities [29,30,31,32]. Researchers can observe, at the moment, stem cell migration, differentiation, and interaction with the surrounding tissue, painting a dynamic picture of their therapeutic roles. The sensitivity of this method ensures that even minute quantities of fluorophore-labeled stem cells can be detected. Additionally, the technique’s flexibility is showcased in its capacity for multiplexing, using multiple fluorophores simultaneously to track different cell populations or processes within the same organism [29,33]. However, no method is without its challenges. Fluorescence imaging grapples with issues like tissue penetration depth, potentially hindering the visualization of stem cells deep within tissues. Photobleaching, where fluorophores lose their fluorescence after extended light exposure, can also challenge long-term observations. Despite its challenges, fluorescence imaging remains a powerful tool in stem cell tracking, offering vivid and invaluable insights into the world of regenerative medicine. In addition, several studies adopted other imaging technologies, such as fluorescence, for multi-imaging systems. 

**Figure 1:**
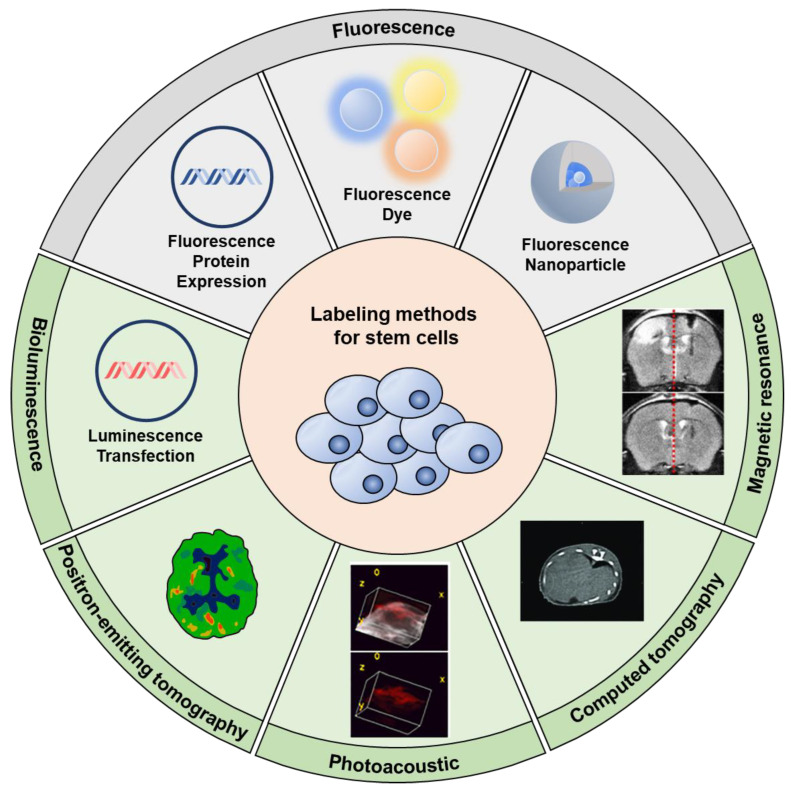
Schematic illustration of strategies for stem cell tracking. Graphical illustration depicts methods for tracking stem cells after implantation. Stem cells can be mono-labeled using fluorescence through fluorescence protein transfection, dye conjugation, and nanoparticle uptake. Fluorescence imaging can be combined with various imaging strategies, such as BL imaging, PET, PA, CT, and MRI, for synergistic detection. The redline on the MR images indicate the midline of the brain. Reproduced with permission from [41,42,43].

To adopt fluorescence techniques for stem cell tracking, various imaging modalities have served as the primary means of rendering these cells visible (Figure 1). Single fluorescence labeling methods largely fall into two categories: direct and indirect. Direct labeling, as the name suggests, entails the incorporation of fluorescent markers, such as dyes or nanoparticles, directly into the cells of interest [34,35,36]. This method has the appeal of simplicity and immediacy; once the cells are labeled, they’re ready to be visualized under the appropriate imaging system. A classic example includes the use of lipophilic membrane dyes, where the dye particles intercalate into the cell membrane, rendering the cell detectable under fluorescence imaging. However, there are concerns associated with direct labeling. Over time, as the cell undergoes metabolic processes or multiplies, the intensity of the fluorescence can diminish. Moreover, there is always an underlying concern about the biocompatibility of the dye or nanoparticle used and whether it might inadvertently alter the functionality or viability of the stem cell. Conversely, indirect labeling leans on advancements in molecular biology, primarily through the genetic modification of stem cells [37,38,39,40]. Here, stem cells are engineered to express fluorescent proteins, typically derived from sources like the jellyfish Aequorea victoria, which naturally emits green fluorescence. The benefit of this method is its longevity. As these genetically modified cells divide, their progeny will also express the fluorescent protein, ensuring consistent tracking over extended periods. Additionally, the risks of label dilution or marker loss are significantly mitigated. However, the complexities of genetic modification, including potential off-target effects or unintended genetic disruptions, need careful consideration. Consequently, researchers could choose appropriate labeling methods for their purpose, considering the pros and cons of each strategy (Table 1).

The realm of stem cell research and therapy heavily relies on imaging techniques to gain insights into cell behavior post-transplantation. While fluorescence imaging stands as a stalwart in this arena, it is not without its drawbacks. Primarily, the depth of tissue penetration in fluorescence imaging is limited [44,45]. As light scatters and is absorbed by biological tissues, the ability to detect fluorescent signals diminishes, especially when trying to visualize deeper tissues. Enter the concept of multimodal imaging: an approach that amalgamates the strengths of different imaging modalities, compensating for the limitations of each. By coupling fluorescence imaging with other techniques, such as magnetic resonance imaging (MRI) or positron emission tomography (PET), researchers can glean a more holistic picture of stem cell behavior in vivo [46,47]. For instance, while fluorescence provides molecular specificity and real-time monitoring capabilities, MRI can offer high-resolution anatomical details, and PET can offer insights into cellular metabolic processes. This harmonized imaging approach is especially vital when considering the multifaceted environments that stem cells navigate within the body. To truly harness the therapeutic potential of stem cells, one must understand their migration patterns, integration into host tissues, differentiation pathways, and potential side effects like tumorigenesis. Multimodal imaging, with its ability to provide a comprehensive amalgamation of anatomical, functional, and molecular data, becomes indispensable in this context. Moreover, the use of multimodal imaging can be thought of as insurance against data loss or misinterpretation. If one modality fails to capture a crucial detail or is hampered by technical challenges, the other modalities can fill in the gaps, ensuring a more accurate and detailed assessment of stem cell behavior.

**Table 1:** Labeling method summarization. Summarization of stem cell tracking methods with mono- and multimodal imaging [48].

Labeling Methods		Pros	Cons	Applicable Cell Type	Tracking Duration
Fluorescence mono-labeled	Protein expression	Genetic labeling Long-term tracking	Affect cell function possibility Time-consuming	Most types	Long-term
	Fluorescence dye	High fluorescence intensity	Photobleaching Possibility of cell toxicity	Broad type but depends on cell toxicity	Short to mid-term
	Nanoparticle	High stability Minimal photobleaching	Affect cell function possibility Possibility of cell toxicity	Broad type but depends on nanoparticle composition	Mid to long-term
Fluorescence combination	BL	High sensitivity No external source required	Low spatial resolution	Most types	Mid to long-term
	PET	High sensitivity Deep tissue imaging	Radioactive tracer High cost	Broad types	Depend on contrast agents
	PA	High resolution Deep tissue imaging	Complex instrumentation Limited contrast agents	Broad range	Depend on contrast agents
	CT	High resolution Deep tissue imaging	Radiation exposure Lower soft tissue contrast	Broad range	Short to mid-term
	MRI	No radiation Excellent soft tissue contrast	High cost Time-consuming	Broad range	Depend on contrast agents

The ideal tracking strategy should provide real-time images for the desired duration. Additionally, the methods used must not disrupt the original function of the stem cells, as any alteration in function post-treatment could render the experiments unreliable. Therefore, in this review, we delve into the transformative role of stem cells in regenerative medicine, emphasizing the necessity of in vivo tracking by introducing several studies that assess changes in stem cell functions, such as cell viability, following tracking modalities [48,49,50]. Among the modalities, fluorescence imaging stands out for its real-time visualization, but its depth limitations necessitate the adoption of multimodal approaches, combining with MRI or PET for comprehensive insights. Direct and indirect stem cell labeling methods further aid in monitoring, ensuring safe and effective therapeutic applications. The synergy of these techniques offers a holistic understanding of stem cell behavior in complex biological terrains.

## 2. Fluorescence Imaging-Based Monomodal Stem Cell Tracking

### 2.1. Fluorescence Protein Expression

Induction of intracellular fluorescence protein expression provides a promising approach to fluorescence imaging-based in vivo stem cell tracking. Fluorescence proteins are synthesized inside the cells via transfection with corresponding genes, which are highly biocompatible without influencing cell viability or functionality [51,52]. Moreover, the techniques for fluorescence protein fusion have been fully established, allowing the selective visualization of certain intracellular components through fluorescence protein tagging [53,54,55]. Over 70 fluorescence proteins with different fluorescence emission wavelengths have been engineered, and their intracellular expression protocols have been developed. Among them, green fluorescence proteins (GFPs) are most broadly exploited for cell labeling due to their strong signals over autofluorescence in physiological conditions and low photobleaching [56,57,58]. W. Tao et al. compared the long-term in vivo tracking performances of enhanced GFP (EGFP)- and Discosoma sp. red fluorescent protein (DsRed)-expressing hematopoietic stem cells (HSCs) in mouse models [59]. The transplanted EGFP-expressing HSCs could survive in recipient mice and repopulate to maintain their fluorescence emission over 15 months. In contrast, DsRed-expressing HSCs could not be preserved after their transplantation and showed a gradual reduction in their fluorescence signals within 3 months, demonstrating the superior labeling efficiency of EGFP. H. Shichinohe et al. labeled bone marrow stromal cells (BMSCs) with GFPs (GFP-BMSCs) to monitor their long-term biodistribution in permanent middle cerebral artery occlusion mouse models [60]. GFP-BMSCs were isolated from the femur bone marrow of EGFP transgenic mice and transplanted into the ipsilateral striatum of cerebral artery-occluded mice, and their in vivo fluorescence imaging was performed for 12 weeks. The transplanted GFP-BMSCs were not visible in the first 2 weeks post-injection since their fluorescence signals could not be detected through the skull. After 4 weeks, however, the fluorescence emission in the right parietal region was observed and became stronger at 12 weeks, indicating the successful in vivo tracking of GFP-BMSC migration to the ischemic area. In another study by A.-K. Hadjantonakis et al., GFPs were fused with human histone proteins in embryonic stem cells (ESs) for the specific labeling of nucleosomes [61]. Histone H2B-tagged EGFP fusion (H2B-EGFP)-expressing ESs were obtained via the transfection of pH2B-EGFP plasmids and electroporation, confirming the consistent transgene expression. Chromosomes in ESs were selectively visualized via fluorescence imaging as H2B-EGFPs were incorporated into chromatin, and H2B-EGFP expression did not affect their mitosis or meiosis. Through the implantation assessment of H2B-EGFP-expressing ESs into mouse blastocysts, it was demonstrated that H2B-EGFP could not only provide the in situ fluorescence images of implanted ESs but also assist in determining their in vivo behavior, possible mutation, and death.

Despite the remarkable labeling efficacy, versatility, and biocompatibility of fluorescence protein expression in stem cell imaging, they have some limitations in their relatively complicated procedures and high costs compared to other labeling methods [62]. In addition, it is difficult to consistently control the expression levels of fluorescence proteins to be similar in all experiments, causing a burden on the quantitative analysis. The genetic modification of stem cells for fluorescence protein expression may provoke genotoxicity or immunogenicity as well.

### 2.2. Fluorescent Dye Labeling

Small molecular dye-based labeling is the most convenient and prompt method to endow in vivo fluorescence trackability to stem cells, wherein stem cells are simply incubated in dye-containing media for less than an hour. Upon incubation, the cell-internalized dyes are supposed to specifically interact with certain cellular components, such as DNA or the cell membrane, depending on their molecular design, to avoid immediate exocytosis [63,64]. Small molecular dyes do not require any genetic modification or the use of endocytosis-enhancing vectors for cell labeling, greatly reducing the labeling cost and complexity [65]. A considerable number of dyes with various fluorescence spectra, including derivatives of cyanine, fluorescein, coumarin, bisbenzimide, and deoxyuridine, are commercially available for cell labeling, and their labeling protocols are also fully established [66,67,68,69]. Chloromethyl-dialkylcarbocyanine (DiI) is a family of carbocyanine dyes and is frequently used for stem cell labeling due to its biocompatibility. DiI preferentially diffuses and binds to cell membranes due to the lipophilic long carbon chains in its molecule, leading to uniform labeling with orange fluorescence throughout the entire cell [70,71]. It was reported that mesenchymal stem cells (MSCs) could be successfully labeled with DiI at a high labeling efficacy, and the DiI stably remained in cell membranes for 6 days without being released or affecting cell viability [72]. F. Ji et al. compared the effectiveness of DiI and GFP for the labeling of human umbilical cord-derived MSCs (hUC-MSCs) and their short-term in vivo tracking [73]. The labeling efficiency of DiI in hUC-MSCs was measured at 95 ± 12.2%, similar to that of intracellularly delivered GFP using adenovirus vectors. After the transplantation into nasal mucosa-injured guinea pigs, DiI-labeled hUC-MSCs were detectable in the injured regions for up to 20 days, although their fluorescence signals gradually decreased, showing comparable efficacy to the viral vector-mediated GFP labeling. 1,1-Dioctadecyl-3,3,3,3-tetramethylindodicarbocyanine (DiD), another family of dicarbocyanine dyes with red fluorescence emission, was also investigated for the labeling and in vivo tracking of human adipose-derived MSCs (haMSCs) in rat osteoarthritis models [74]. haMSCs preserved their proliferation and differentiation abilities without any deterioration after DiD labeling. The fluorescence signals by DiD-labeled haMSCs were observable for 10 weeks in osteoarthritis-induced knee joints when they were injected intra-articularly, while no fluorescence was visible in other organs due to their specific localization. Meanwhile, J. Chen et al. synthesized a novel quinoxalinone-based near-infrared (NIR) fluorescent dye (QSN) and used it for human neural stem cell (hNSC) labeling [75]. QSN strongly targeted cell membranes while barely binding to other organelles, and it exhibited intensive NIR emission, outstanding photostability, and photobleaching resistance. QSN-labeled hNSCs showed high viability and long-term fluorescence visibility for at least 6 weeks after their transplantation into the mouse brain striatum. Notably, QSN-labeled hNSCs expressed in vivo fluorescence signals for a much longer period compared to conventional carbocyanine dye-labed ones, validating the superior photobleaching resistance of QSN. Other fluorescent dyes, including carboxyfluorescein succinimidyl ester (CFSE) and indocyanine green (ICG), have also been employed for stem cell labeling and demonstrated their applicability in stem cell tracking in vivo, wherein their label durability was slightly different depending on their molecular structures and corresponding cell binding affinity [76,77,78].

### 2.3. Fluorescent Nanoparticle Labeling

The main hurdle of fluorescent dye-based stem cell labeling is attributed to its short labeling duration and rapid photobleaching [79]. Small molecular dyes would be immediately effluxed, metabolized, and lose their functionality inside the cells, making it hard to achieve long-term tracking of stem cells in vivo. Fluorescent nanoparticles can be a good option for stem cell labeling, addressing the drawbacks of small molecular fluorescent dyes and even fluorescent proteins. Fluorescent nanoparticle-based stem cell labeling can ensure prolonged fluorescent labeling with high photostability over small molecular dye-based methods due to the stable and bulky nanoparticle structures while alleviating the complications in terms of labeling cost and process. Various types of fluorescent nanoparticles have been investigated for in vivo stem cell tracking, which were introduced in this chapter, and their characteristics and advantages were addressed.

The use of fluorescent dye-embedded nanoparticles is the simplest way for nanoparticle-based stem cell labeling, which can improve the stability of fluorescent dyes inside the cell. Various methods and components have been proposed to fabricate dye-embedded nanoparticles for stem cell labeling, wherein fluorescent dyes can be either encapsulated, physically adsorbed, or chemically conjugated to organic/inorganic nanoparticles via different mechanisms [80,81]. For example, B. F. Morgharbel et al. explored the labeling of adipose-derived MSCs (ADMSCs) with curcumin-loaded polycaprolactone nanoparticles (NPCs) for fluorescence imaging-based stem cell tracking [82]. NPCs with 189.4 nm size and −0.112 mV zeta potential were manufactured through the nanoprecipitation method, and curcumin was physically entrapped inside the NPCs with 99.8% encapsulation efficiency, wherein the loaded curcumin was supposed to act both as a fluorescent dye and as a therapeutic for ADMSC survival enhancement. NPCs provoked ignorable cytotoxicity against ADMSCs at a high concentration of up to 30 μM and were effectively taken up to express prolonged fluorescence signals inside the cells over 8 days. Through in vivo fluorescence imaging, the migration of NPC-labeled ADMSCs to infarcted myocardial tissues was observed after their subcutaneous transplantation. In another study by H. Bao et al., 6-carboxyfluorescein (FAM) dye-attached gold nanoparticles were developed as both tracers and sensors to label hMSCs and detect the intracellular expression of hepatocyte growth factor (HGF) mRNA [83]. FAMs were conjugated with HGF mRNA-recognizing oligonucleotide sequences, and gold nanoparticles were decorated with complementary sequences. Those FAMs and gold nanoparticles were then formed into complexes via the specific physical interactions between two oligonucleotide sequences, obtaining nanoflare tracers. FAMs did not express fluorescence signals upon being attached to the nanoflare tracers due to the fluorescent resonance energy transfer (FRET) effect and were detached from the tracers to recover the fluorescence in the presence of HGF mRNAs. The nanoflare tracers were successfully endocytosed into hMSCs and visualized the HGF mRNA expression with high sensitivity. The in vivo assessment with pulmonary fibrosis mouse models discovered that the transfer of nanoflare tracer-labeled hMSC into fibrotic lung tissues was traceable through in vivo fluorescence imaging. Research on stem cell labeling with polymeric nanoparticles covalently bonded to fluorescent dyes (fluoNPs) was also reported elsewhere [84]. Rhodamine B (RhB) dye-conjugated 2-hydroxyethyl methacrylate (HEMA) macromonomer was synthesized and co-polymerized with methyl methacrylate (MMA) via emulsion polymerization to acquire fluoNPs with a 93.57 nm size and −20.60 mV zeta potential. The RhB content in FluoNPs was controlled to 0.1% (*w*/*w*). The fluoNP-labeled human amniotic fluid cells (hAFCs) stably emitted fluorescence for 3 days without changes in their biological features, confirming the effectiveness of fluoNP labeling. In another study by S. Liu et al., fluorescent polymeric nanoparticles composed of di(thiophene-2-yl)-diketopyrrolopyrrole (DPP) dye-conjugated PCL (PCL-DPP-PCL) were examined for the labeling of human MSCs (hMSCs) [85]. PCL-DPP-PCL nanoparticles exhibited high photostability and biocompatibility, remaining inside hMSCs and continuously emitting fluorescence for 4 weeks.

To extend the in vivo traceable period of nanoparticle-labeled stem cells, it is necessary to increase the cellular uptake efficiency and intracellular retention of fluorescent nanoparticles while minimizing their cytotoxicity. Through the surface modification of fluorescent nanoparticles with additional moieties such as positively charged molecules, cell-penetrating peptides (CPPs), and cell-binding ligands, the endocytosis of fluorescence nanoparticles can be greatly enhanced. D. Yeo et al. utilized poly-L-lysine (PLL)-modified poly(lactic-co-glycolic acid) (PLGA) nanoparticles as a nanosensor platform for stem cell tracking and observation of intracellular biological function [86]. Notably, the PLL-PLGA nanosensors were designed to possess positive surface charges with the PLL coat and large particle sizes (500–1000 nm) to achieve higher labeling efficiency and prolonged intracellular retention. The PLL-modified nanosensors exhibited improved cellular uptake compared to the unmodified ones, as confirmed by ~3-fold enhanced intracellular fluorescence intensity. Three different types of biosensing molecules that emit fluorescence signals in response to specific biomarkers were loaded into the PLL-PLGA nanosensors and slowly released from the cell-uptake nanosensors for 28 days to detect intracellular biomarkers. When hMSCs were labeled with PLL-PLGA nanosensors, they were traceable via fluorescence imaging, and even their intracellular biomarker expression, such as esterase, nitric oxide, and β-actin mRNA, was detectable as well. In addition, the effect of nanoparticle surface charges on stem cell labeling was investigated in detail by T.-H. Chung et al. [87]. Rhodamine B isothiocyanate (RITC)-containing mesoporous silica nanoparticles (MSNs) were modified with different amounts of positively charged *N*-trimethoxysilylpropyl-*N*,*N*,*N*-trimethylammonium chloride (TMAC) to diversify their surface charges from −4.90 to +19.0 mV, and hMSCs were incubated with them to assess their cellular uptake and cytotoxicity. For the precise comparison, the sizes (108–115 nm) and fluorescence emission of RITC-MSNs were consistently regulated regardless of their surface charges. In the cellular assay with hMSCs, the endocytosis ratio of RITC-MSNs tended to increase as they held stronger surface positive charges. Surface charges of endocytosed RITC-MSNs did not affect the viability and functionality of hMSCs, concluding that RITC-MSNs with higher surface positive charges were more effective for stem cell labeling. Other fluorescent dye-embedded nanoparticles with positive surface charges, including polyethyleneimine (PEI)-coated core-shell fluorescent silica nanoparticles and cationic niosomes composed of Span80 and 1,2-dioleoyl-3-trimethylammonium-propane (DOTAP), have been reported elsewhere, verifying their improved stem cell labeling efficacy [88,89].

The surface modification of nanoparticles with CPPs is another common strategy to increase cell labeling efficiency of fluorescence nanoparticles, wherein Tat peptide has been broadly adopted the most. Y. Yuan et al. applied Tat peptide-conjugated fluorescent polymer dots (Tat-Pdots) in fluorescence imaging-guided tracking of MSCs in vivo [90]. NIR fluorescence-emitting Pdots were fabricated via the co-encapsulation of two different semiconducting polymers (DFDBT and NIR800) with poly(styrene-co-maleic anhydride) (PSMA) copolymers to secure their physiological stability, and the Pdots were further surface-modified with Tat peptides. Tat-Pdots were vigorously endocytosed into MSCs with the action of Tat peptides on their surface, leading to ~200-fold higher fluorescence brightness of Tat-Pdot-labeled MSCs compared to that of unmodified Pdot-labeled ones. Tat-Pdot labeling did not decrease the viability of MSCs during 4 h incubation at a concentration of up to 40 μg/mL. Throughout 7 days of in vivo fluorescence monitoring, the localization tendency of Tat-Pdot-labeled MSCs could be visualized. The superior stem cell labeling property of Tat-conjugated fluorescence nanoparticles was further proven by G. Jin et al. [91]. Fluorescence-emitting conjugate polymers (poly(9,9-dioctylfluorene-alt-benzothiadiazole; PFBT)) were encapsulated with 1,2-Distearoyl-sn-glycero-3-phosphoethanolamine-N-[maleimide-PEG 5000] (DSPE-PEG-Mal) and DSPE-PEG-microRNA-1 (miR-1) via nanoprecipitation and successively modified with Tat peptides to produce Tat-miR-1-PFBT nanoparticles. The labeling efficiency of Tat-miR-1-PFBTs against MSCs was compared to that of Qtracker 585, a conventional quantum dot-based labeling probe. Tat-miR-1-PFBTs showed high labeling stability; thereby, the fluorescence signal from Tat-miR-1-PFBT-labeled MSCs could be observed for 20 days, whereas Qtracker 585-labeled MSCs gradually lost their fluorescence emission within 10 days. The percentages of remaining labels in Tat-miR-1-PFBT- and Qtracker 585-labeled MSCs after 5 days were 90 and 50%, respectively, confirming the durability of Tat-miR-1-PFBT labeling. The long-term in vivo tracking of Tat-miR-1-PFBT-labeled MSCs was also available through fluorescence imaging, wherein the localization of MSCs in infarcted myocardial tissues was observed for 14 days. Moreover, Tat-miR-1-PFBTs favorably transfected miR-1, a cardiac differentiation-enhancing microRNA, into MSCs with high efficiency, simultaneously performing cell labeling and gene delivery.

**Figure 2:**
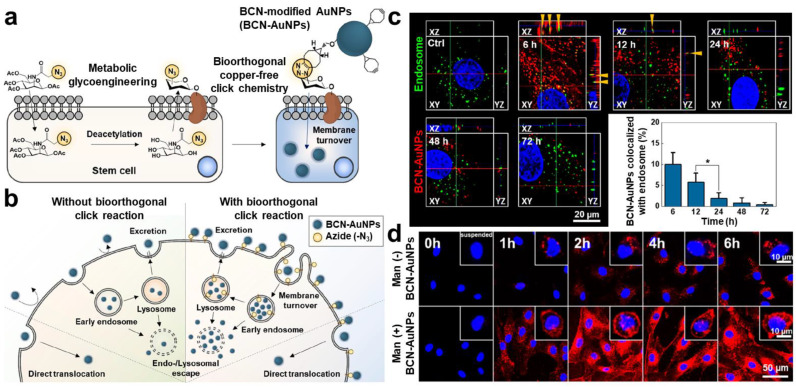
Fluorescent nanoparticle-based stem cell labeling via metabolic glycoengineering-involved biorthogonal click chemistry. Schematic illustrations depict (**a**) the mechanism of stem cell labeling via glycoengineering-involved bioorthogonal chemistry and (**b**) the endocytosis of BCN-AuNPs labeled on the cell membranes. (**c**) In vitro confocal fluorescence images of BCN-AuNP-labeled hMSCs showing the intracellular distribution of BCN-AuNPs. The orange arrows indicate colocalized fluorescence of BCN-AuNPs and lysosome. (**d**) Confocal fluorescence images of BCN-AuNP-labeled hMSCs with or without the preceding Ac4Man-NAz treatment. (*) indicate difference at the *p* < 0.05 significance. Reproduced with permission from [49]. ACS Publications, 2021.

In the meantime, S. Lim et al. proposed a novel approach to increase the efficiency of nanoparticle-based stem cell labeling and improve the label duration through the application of metabolic glycoengineering and bioorthogonal click chemistry [92]. For the cell labeling, bicyclo[6.1.0]nonyne (BCN)-modified and Cyanine 5.5 (Cy5.5) dye-conjugated glycol chitosan nanoparticles (BCN-CNP-Cy5.5s) and three different azide-containing metabolic precursors were separately prepared. hMSCs were previously incubated with azide-containing precursors to induce azide expression on the plasma membranes, and BCN-CNP-Cy5.5s were subsequently treated with hMSCs for labeling via a copper-free click reaction between the BCN and azide. Comparing the azide expression efficiency of hMSCs after incubating with three precursors, respectively, hMSCs incubated with tetraacetylated *N*-azidoacetyl-D-mannosamine (Ac_4_Man-NAz) showed the highest azide expression level on their membrane. hMSCs were successfully labeled with BCN-CNP-Cy5.5s upon the preceding Ac_4_Man-NAz incubation, showing higher labeling efficiency and longer labeling preservation compared to those without Ac_4_Man-NAz incubation or BCN-Cy5.5 labeling. Importantly, the hMSCs labeled with both Ac_4_Man-NAz and BCN-CNP-Cy5.5 were detected at their injected sites over 15 days through in vivo fluorescence imaging, whereas those labeled with BCN-CNP-Cy5.5s were not visible after 5 days post-injection. In another study by the same research group, BCN- and Cy5.5-conjugated gold nanoparticles (BCN-AuNP-Cy5.5s) and Ac4Man-NAz were also evaluated for fluorescence labeling of hMSCs (Figure 2a) [49]. Interestingly, it was observed that BCN-AuNP-Cy5.5s labeled on hMSCs via glycoengineering-assisted bioorthogonal click chemistry were not only located on the cell membranes but also visible inside the cells, which was considered to extend the label persistence. The specific endocytosis mechanism of ectocellularly labeled BCN-AuNP-Cy5.5s was discovered, wherein the multivalent binding of BCN-AuNP-Cy5.5s to azides on the membrane caused the membrane turnover to internalize BCN-AuNP-Cy5.5s via endosome formation (Figure 2b) [93]. Endosome-mediately endocytosed BCN-AuNP-Cy5.5s underwent endosomal escape, and eventually, a large amount of them was localized in the cytosol (Figure 2c). The gradual endocytosis of bioorthogonally labeled BCN-AuNP-Cy5.5s over time was detected through in vitro fluorescence imaging, resulting in a 3.0-fold stronger intracellular fluorescence intensity compared to that without the preceding Ac_4_Man-NAz treatment after 6 h post-incubation (Figure 2d). The labeling efficiency of BCN-AuNP-Cy5.5 with Ac_4_Man-NAz was also higher than that of small-molecule dye-based bioorthogonal labeling since small molecular dyes cannot be internalized into cells via multivalent binding and membrane turnover. The results of two series of studies indicated that metabolic glycoengineering and click chemistry-based cell labeling are powerful methods to enhance labeling efficiency in stem cell imaging.

Quantum dots (QDs) are semiconducting inorganic nanocrystals emitting strong fluorescent signals with high photostability. Due to their versatile tunability of fluorescence emission spectra depending on their compositions and sizes, which is hard to acquire with organic dyes, QDs have been extensively employed for multiplex bioimaging [94,95,96]. Several studies have been carried out on the use of QDs in stem cell labeling. B. J. Muller-Borer et al. labeled rat bone marrow MSCs with CdSe/ZnS core/shell QDs sized to 10–15 nm and investigated the effect of QDs on MSCs [97]. QDs were taken up by MSCs, aggregated inside the MSC vesicles around nuclei, and remained intracellularly for up to 120 h. The incubation of MSCs with 20 nmol/L QDs for 24 h did not reduce the cell viability and neither DNA damage nor decreased cell proliferation were observed. Meanwhile, S. Lin et al. conducted the labeling of murine ESs with six different QDs using Qtracker^TM^ to compare their labeling efficiency [98]. None of them affected the viability, proliferation, and differentiation of ESs at concentrations of 10 nM. When subcutaneously transplanting ESs onto the backs of mice after the labeling with six QDs, respectively, Q800-labeled ESs showed the strongest fluorescence intensity with a prolonged duration of 14 days. In another study by G. Chen et al., Ag_2_S QDs were adopted for the real-time tracking of intravenously administered hMSCs in cutaneous injury mouse models [99]. The slow migration of Ag_2_S QD-labeled hMSCs to injured regions for wound healing could be visualized for 30 days via QD-based NIR-II imaging with high spatiotemporal resolution, and the stimulated migration in response to stromal cell-derived factor-1a (SDF-1a) chemokine treatment was also detectable. The proliferation, gene expression, and differentiation of hMSCs did not meaningfully change after the Ag2S QD labeling, successfully preserving with their wound-healing properties.

**Figure 3:**
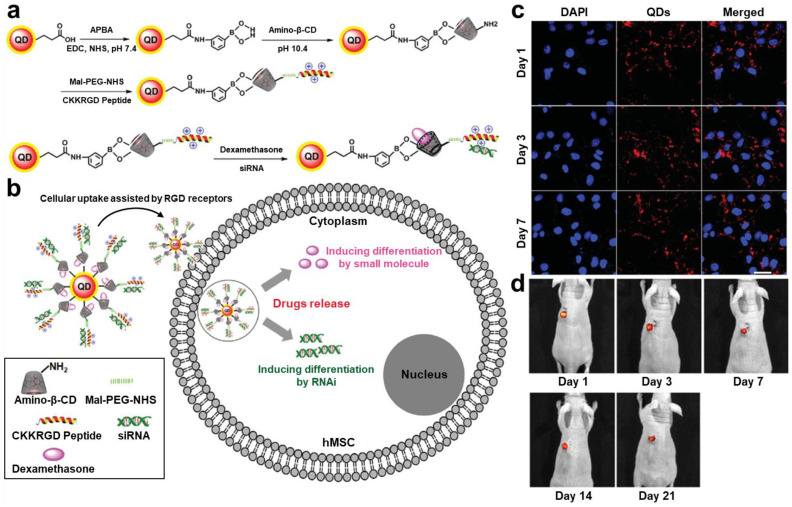
Quantum dot-based fluorescence labeling probe. (**a**) Synthetic scheme for preparing RGD-β-CD-QDs. (**b**) Schematic illustration for the mechanism of action of RGD-β-CD-QDs. (**c**) In vitro confocal fluorescence images of RGD-β-CD-QD-labeled hMSCs showing the intracellular retention of RGD-β-CD-QDs. (**d**) In vivo long-term fluorescence images for tracking the RGD-β-CD-QD-labeled hMSCs after their subcutaneous transplantation. Reproduced with permission from [101]. Wiley, 2016.

Similar to fluorescent dye-embedded nanoparticles, it is important to enhance the endocytosis and extend the intracellular retention of QDs for efficient in vivo stem cell tracking. Y. Lei et al. fabricated Tat peptide-conjugated QDs (QD-Tat peptides) for the sufficient labeling of MSCs [100]. QD-Tat peptides were produced by coating CdSe/ZnS QDs with DSPE-PEG 2000 amine and conjugating cysteine-modified Tat peptide to the end of the PEG chain. QD-Tat peptides were actively taken up by MSCs and localized within perinuclear spaces, while QDs without Tat peptide conjugation showed no significant cellular internalization. The endocytosis efficiency of QD-Tat peptides was over 95%, which promoted the tracking of QD-Tat peptide-labeled MSCs via fluorescence microscopy after intravenous administration to mice. Arg-Gly-Asp (RGD) peptides have also been utilized to improve the labeling efficiency of QDs [101]. J. Li et al. synthesized RGD peptide- and β-cyclodextrin (CD)-modified CdTe/ZnS QDs (RGD-β-CD-QDs) and employed them for human MSC (hMSC) labeling (Figure 3a). The surface-conjugated RGD peptides assisted the cellular uptake of RGD-β-CD-QDs by mediating the interaction with RGD receptors, and β-CDs improved the hMSC viability after the labeling (Figure 3b,c). In the in vivo assessment, the long-term tracking of RGD-β-CD-QDs-labeled MSCs after their subcutaneous implantation was accomplished for up to 21 days without significant reduction in fluorescent signals (Figure 3d).

Despite the feasible tunability and high photostability of QDs, their high toxicity attributed to the use of heavy metals severely restricts their actual application in stem cell labeling. In this regard, carbon-based QDs with higher biocompatibility have been developed and investigated for stem cell labeling, replacing metal-based QDs. T. Malina et al. labeled adipose tissue-derived hMSCs with quaternized carbon dots (QCDs) and analyzed their in vivo fluorescence imaging [102]. QCDs were synthesized via thermal oxidation of organic salts, showing a narrow size distribution (2–4 nm) and desirable colloidal stability with quaternary ammonium-coated surfaces. They emitted red fluorescence light at 600 nm wavelength under 490–520 nm light irradiation and presented high biocompatibility at a concentration of up to 100 μg/mL. QCD-labeled hMSCs expressed strong and long-lasting fluorescence signals when they were subcutaneously or intravenously transplanted into tumor-bearing immunodeficient mice, promoting the monitoring of their accumulation in tumor tissues for 2 weeks. In another study by M. Zhang et al., water-soluble graphene quantum dots (GQDs) were produced through the electrolysis and reduction of graphite in mild conditions and used in stem cell labeling as well [103]. GQDs emitted strong yellow fluorescence with a high quantum yield (~14%), and their dispersion stability in aqueous media was secured via their edge modification with hydrazide groups. When stem cells were incubated with GQDs, the cells were viable at high GQD concentrations of 50 μg/mL, while bare semiconducting QDs induced strong cytotoxicity even at much lower concentrations. GQD-labeled stem cells exhibited bright fluorescence with 405 nm light excitation, confirming the potency of GQD in stem cell tracking applications. On the other hand, the effect of surface charges on the labeling efficiency and cytotoxicity of carbon-based QDs was evaluated against hUC-MSCs [104]. As the surface positive charge increased from −6.78 to +23 mV, carbon-based QDs exerted higher labeling efficiency and brighter fluorescence signals, but their cytotoxicity was also elevated. It was found that carbon-based QDs with moderate positive charges (+4.12 mV) were preferable for stem cell labeling, showing high biocompatibility and sufficient labeling efficiency.

Nanodiamonds are carbon-based fluorescent nanomaterials emerging as other alternatives to QDs with strong toxicity due to their heavy metal content [105,106,107]. They are composed of sp^3^-carbons doped with negatively charged nitrogen-vacancy centers, which makes them highly biocompatible and red-light fluorescent (600–800 nm) [108]. Nanodiamonds also have remarkable chemical inertness and photostability in physiological conditions, showing desirable properties for application in biological imaging. The studies on nanodiamond-based stem cell labeling have been mainly conducted by H.-C. Chang and colleagues, wherein various cancer or normal stem cells were examined for their in vivo tracking [109,110]. Fluorescent nanodiamonds (FNDs) with ~100 nm in size were fabricated via radiation to type Ib diamond powders and taken up by lung stem cells (LSCs) for labeling [111]. FND-labeled LSCs expressed far-red fluorescence in vitro, ~45-fold higher signals than unlabeled cells. FNDs did not harm the functionality or viability of LSCs and remained intracellularly for a prolonged period of up to 15 days without significant exocytosis. After the intravenous administration to lung-injured mice, the lung accumulation and engraftment of FND-labeled LSCs were observable via fluorescence imaging. The labeling of quiescent cancer stem cells (CSCs) using FNDs was also performed in another study by the same researchers [112]. FND labeling was determined not to induce DNA damage or suppress cell proliferation, and it showed a longer tracking duration (~20 days) compared to conventional staining probes such as 5-ethynyl-2′-deoxyuridine (EdU) and carboxyfluorescein diacetate succinimidyl ester (CFSE). In the mammosphere-forming efficiency assessment, FND labeling facilitated clearly distinguishing the slow-proliferating/quiescent CSCs, demonstrating its effectiveness for stem cell tracking. Moreover, L.-J. Su et al. labeled human placenta choriodecidual membrane-derived MSCs (pcMSCs) using FNDs for their tracking in miniature pigs [113]. FNDs were coated with human serum albumins (HSAs) to prepare HSA-FNDs, which improved their media dispersity. When HSA-FND-labeled pcMSCs were intravenously administered to miniature pigs, their distribution in organs was precisely detectable through fluorescence imaging, even enabling the quantification of transplanted cells.

Upconversion nanoparticles (UCNPs) are potent materials for in vivo stem cell imaging as they elicit unique properties that cannot be achieved with other fluorescent dyes or particles. UCNPs absorb two or more photons with long wavelengths (low energy) like near-infrared (NIR) lights and emit a photon with short wavelengths (higher energy), called anti-Stokes shift, thereby enabling the use of longer wavelength light sources for deep tissue imaging [114,115]. In addition, the emission of light with short wavelengths by UCNPs promotes more precise imaging in vivo since upconversion luminescence (UCL) can be conveniently identified over autofluorescence by biocomponents [97,116]. L. Zhao et al. utilized the (α-NaYbF_4_:Tm^3+^)/CaF_2_ UCNPs for the labeling of rat MSCs (rMSCs) and compared the differentiation behavior of rMSCs before and after the labeling [117]. Positively charged PEI was covalently conjugated on the surface of (α-NaYbF_4_:Tm^3+^)/CaF_2_ UCNPs (PEI-UCNPs) to improve their cellular uptake. rMSCs were successfully labeled with PEI-UCNPs, and the cell-internalized PEI-UCNPs were not leaked out of rMSCs over 14 days. The viability of rMSCs was not significantly impaired even with 4 h exposure to PEI-UCNPs at up to 100 μg/mL, nor was their osteogenic or adipogenic differentiation. In another study by C. Wang et al., NaYF_4_ UCNPs were synthesized and coated with amine-PEG-grafted poly(maleic anhydride-alt-1-octadecene) (C_18_PMH-PEG-NH_2_) to prepare UCNP-PEGs for their application to mouse MSC (mMSC) labeling [118]. UCNP-PEGs were further conjugated with oligo-arginine (UCNP-PEG-ARGs) to enhance endocytosis, which resulted in their highly positive surface charges (+30.2 mV) and ~10-fold increased labeling efficiency compared to UCNP-PEGs without oligo-arginine. UCNP-PEG-ARGs labeling did not affect the viability or differentiation capability of mMSCs at their concentrations up to 0.2 mg/mL, maintaining their long-term intracellular fluorescence over 10 days. When UCNP-PEG-ARG-labeled mMSCs were subcutaneously transplanted to mice, they could be detected with ultra-high sensitivity even at a low cell amount of ~10 due to the UCL of UCNPs. Moreover, UCNP-PEG-ARG labeling enabled the observation of intravenously administered mMSCs to translocate from lung to liver for 24 h.

Attributed to the anti-stokes shift upconverting properties, UCNPs would elicit light-responsive drug-releasing properties in addition to fluorescent imaging. J. Li et al. designed multifunctional UCNPs by coating NaYF_4_:Yb/Tm UCNPs with silica (SiO_2_) and modifying the surface with Cys-Arg-Gly-Asp (CRGD) peptides and kartogenin (KGN)-conjugated UV-cleavable linkers, producing RGD-KGN-UCNP@SiO_2_s for tracking and inducing controlled differentiation of human MSCs (hMSCs) [119]. RGD-KGN-UCNP@SiO_2_s were supposed to be vigorously endocytosed via the action of RGD sequences and release KGNs, differentiation-inducing agents, under NIR irradiation, which would be converted into UV light by UCNPs. hMSCs incubated with RGD-KGN-UCNP@SiO_2_s were not only visualized via fluorescent imaging but also differentiated into chondrocytes to recover the damaged cartilage. The RGD-KGN-UCNP@SiO_2_-labeled hMSCs were detectable in vivo for a long period of up to 28 days after their subcutaneous implantation with methacrylate hyaluronic acid (MeHA) hydrogels. A similar but advanced UCNP-based system for both monitoring and differentiation control of hMSCs was developed by the same research group [120]. This system, called UCNP-Peptide-AIE-siRNA, was to undergo RGD-mediated active endocytosis by hMSCs and release siRNAs to trigger the osteogenic differentiation of hMSCs in response to NIR irradiation. Notably, the endocytosed UCNP-Peptide-AIE-siRNAs were further cleaved by matrix metallopeptidase 13 (MMP13) overexpressed in differentiated osteoblastic cells and aggregated inside the cell, facilitating the distinguished detection of hMSC differentiation through aggregation-induced emission (AIE).

## 3. Fluorescence Imaging-Based Multimodal Stem Cell Tracking

Although fluorescence labeling is determined to offer a non-invasive and practical way to monitor the long-term profile of stem cells in vivo, the transplanted stem cells cannot be fully analyzed via its monomodal use due to inherent or technical limitations. Fluorescence images only show where the labeled stem cells are located by detecting their light emission, implying that anatomical information cannot be obtained with fluorescence imaging [121,122]. In addition, its imaging accuracy would be drastically dropped in deep tissues since the light sources for fluorescence probe excitation usually have limited tissue penetration depth [123,124]. Therefore, the application of multiple imaging modalities is necessary to compensate for the drawbacks of fluorescence imaging and improve the reliability of stem cell tracking. Several different imaging modalities, including bioluminescence (BL) imaging, positron-emitting tomography (PET), photoacoustic (PA) imaging, computed tomography (CT), and magnetic resonance (MR) imaging, have been employed for fluorescence imaging-combined multimodal tracking of stem cells in vivo, which brought different advantages depending on the characteristics of imaging modalities [125]. For instance, in the case of nanoparticle combinations, types of nanoparticles, such as magnet-based ones for MRI or metal-based ones for CT, can be conjugated with or encapsulated in a fluorescence dye for combined tracking. The research cases on fluorescence imaging-based multimodal stem cell tracking are classified according to the additional imaging modalities, and their outcomes are briefly elucidated below, focusing on the beneficial points of multimodal imaging.

### 3.1. Fluorescence-BL Dual Imaging

**Figure 4:**
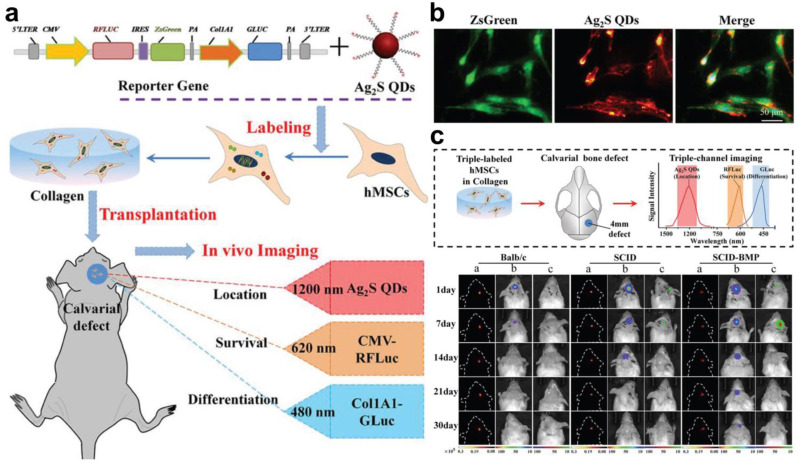
Fluorescence-BL bimodal stem cell labeling. (**a**) Schematic illustration showing the process for the fluorescence-BL dual labeling of hMSCs using luciferase-expressing reporter genes and QDs. (**b**) Confocal fluorescence images of dual-labeled hMSCs exhibiting their fluorescence and BL expression. (**c**) Long-term in vivo images of hMSCs after their transplantation into skull defects (a = fluorescence by QDs, b = BL by RFLuc, c = BL by GLuc). Reproduced with permission from [132]. Wiley, 2018.

To acquire better outcomes in stem cell therapy, it is required to identify not only the biodistribution and localization of transplanted stem cells but also their conditions, such as survival, proliferation, and differentiation. Fluorescence probes can be designed to provide either locational or functional indications of transplanted stem cells, but it is difficult to obtain both information with high accuracy using only a single imaging modality, and multimodal imaging is of great necessity. BL imaging is an imaging modality that emits luminescence by converting chemical energy into light, exerting high sensitivity at a low probe concentration [126,127]. Since BL imaging probes can be tagged on specific intracellular components or organelles and visualize their expression, interaction, and elimination, they are suitable for the imaging of cellular biology [128,129,130]. Moreover, BL imaging gives more precise analysis data in deep tissues compared to fluorescence imaging, as it does not require external light irradiation for excitation [131]. When BL imaging modalities are combined with fluorescence imaging in stem cell tracking, the location and status of stem cells can be conveniently and simultaneously analyzed in vivo. D. Huang et al. conducted the second NIR (NIR-II) fluorescence and BL dual labeling of hMSCs to monitor their location, survival, and differentiation after their transplantation into calvarial defect mouse models [132]. The fluorescence and BL labeling were separately carried out on hMSCs by transfecting them with lentiviral vectors containing red firefly luciferase (RFLuc), Zoanthus sp. GFP (ZsGreen), and Gaussia luciferase (GLuc) genes and subsequently incubating them with Tat peptide-conjugated Ag_2_S QDs (Tat-Ag_2_S QDs) (Figure 4a). Each imaging probe was labeled for different purposes, wherein Ag_2_S QDs, RFLuc, and GLuc were supposed to visualize the biodistribution, viability, and differentiation of transplanted hMSCs, respectively. In vitro assessment of fluorescence and BL dual-labeled hMSCs discovered that hMSCs maintained their viability and functionality after all labeling processes, and Tat-Ag_2_S QD labeling did not affect the expression of RFLuc and Gluc (Figure 4b). The labeled hMSCs were cultured in a collagen matrix and subsequently grafted onto the calvarial defect in mouse models, wherein their retention and osteogenic differentiation behaviors were clearly detected via fluorescence and BL imaging (Figure 4c). The fluorescence and BL dual imaging of stem cells using one multifunctional nanoparticle were also examined elsewhere [133]. Firefly luciferase-conjugated FNDs (Luc-FNDs) were developed to achieve more simple bimodal imaging of stem cells by circumventing the procedural complexity of luciferase gene transfection. The fabricated Luc-FNDs were measured to be 107 nm in size and emitted both fluorescence and luminescence signals inside placenta choriodecidual membrane-derived MSCs (pcMSCs) over 10 days without presenting crucial cytotoxicity. The fluorescence and BL images of recipient mice subcutaneously administered with Luc-FND-labeled pcMSCs showed bright dual signals at the injected area, accomplishing the multiplex imaging of transplanted pcMSCs with Luc-FND labeling.

### 3.2. Fluorescence-PET Dual Imaging

PET is a molecular bioimaging modality showing the accumulation of radioisotope probes in sites of interest [134]. When the radioisotope-tagged biomolecule tracers are injected, the tracers are transported to target tissues and metabolized to induce radioisotope expression in the tissues. Radioisotopes expressed on target tissues emit positrons during their β^+^ decay, and emitted positrons undergo electron–positron pair annihilation to emit γ-rays, which would be recognized by the PET scanner [135]. The imaging durability of PET is determined by the half-life of the radioisotope, wherein ^18^F is frequently used in clinics due to its moderate half-life (~110 min). PET imaging elicits highly sensitive and quantitative features as it uses γ-rays with high energy for detection and visualizes specific substrates radiolabeled at molecular or subcellular levels [135,136]. The fluorescence-PET dual labeling of stem cells allows their precise and systemic in vivo tracking with strong sensitivity, effectively mitigating the tissue penetration issue of fluorescence imaging. S. Gaedicke et al. attempted to detect the tumor stem cells in xenograft glioma tissues using both fluorescence and PET imaging probes since tumor stem cells play a key role in tumor progression, metastasis, and relapse [47]. The fluorescence and PET imaging probes were separately produced by conjugating Alexa 680 dye (Alexa 680-AC133 mAb) or ^64^Cu-NOTA radiopharmaceutical (^64^Cu-NOTA-AC133 mAb) to the AC133 antibody, which was designed to bind to tumor stem cell-overexpressing AC133 antigen. Upon their intravenous administrations, the tumor-targeted accumulation of both Alexa 680-AC133 mAb and ^64^Cu-NOTA-AC133 mAb was observed via fluorescence and PET imaging. The specific binding of AC133 mAb-conjugated probes to cancer stem cells was further validated through histological analysis. T. T. Pham et al. synthesized a PET-fluorescence dual labeling agent (^124^I-FIT-(PhS)_2_Mal) composed of fluorescein dye, ^124^I radioisotope, and dithiophenolmaleimide ((PhS)_2_Mal) moiety, and examined them for in vivo cell tracking [137]. (PhS)_2_Mal moiety was employed for the binding of ^124^I-FIT-(PhS)_2_Mal on cell membrane proteins, and ^124^I radioisotope was chosen due to its long half-life (4.2 days), which assured persistent PET imaging. ^124^I-FIT-(PhS)_2_Mal successfully labeled various types of cells with high efficiency, which was confirmed by bright fluorescence signals mainly found on the cell membranes. The biodistribution of intravenously injected Jurkat cells after their labeling with ^124^I-FIT-(PhS)_2_Mal was evaluated via fluorescence and PET imaging, effectively visualizing the migration of labeled Jurkat cells over 7 days. Yet, few studies on the application of fluorescence-PET bimodal imaging in stem cell tracking have been conducted, even if several cases applying it in cancer diagnosis or immune cell tracking are found, requiring further investigation in the future [138,139,140].

### 3.3. Fluorescence-PA Dual Imaging

PA imaging is a hybrid imaging modality that detects ultrasound signals created by light input. When tissues or organs are illuminated with a short light pulse, a temporary increase in local temperature is induced, and thermoelastic expansion occurs, emitting ultrasound waves [141,142]. Differently from fluorescence, BL, or PET imaging, PA imaging can draw on the features of both molecular and anatomic imaging modalities [143,144]. It enables obtaining localized images with high specificity and contrast by using PA agents. At the same time, it exhibits high sensitivity at the millimeter level due to the expression of ultrasound signals with low scattering [145]. Therefore, the low spatial resolution and sensitivity of fluorescence imaging can be alleviated via fluorescence-PA dual imaging. In addition, one of the most attractive benefits of fluorescence-PA bimodal stem cell imaging is that several organic dyes or nanoparticles are revealed to act not only as fluorescent dyes but also as PA agents, thereby enabling dual imaging without additional labeling [146,147,148]. M. Filippi et al. exploited ICG dyes for fluorescence-PA bimodal tracking of MSCs [149]. MSCs were labeled with ICGs through simple uptake, which showed 2.4 ± 0.5% labeling efficiency after 1-h incubation and persistent intracellular retention of endocytosed ICGs for 7 days. ICG-labeled MSCs expressed both fluorescence and PA signals under the irradiation of 710–760 nm light and a 680–960 nm laser pulse, respectively. When ICG-labeled MSCs were intramuscularly grafted into normal mice, their deposited regions could be visualized through fluorescence-PA dual imaging for 4 days. 1,10-dioctadecyl-3,3,30,30-tetramethylindotricarbocyanine-iodide (DiR) dyes were also reported as fluorescence-PA dual imaging probes for stem cell tracking [150]. Lipophilic DiR dyes were physically inserted into the plasma membranes of MSCs upon their incubation and expressed deep red fluorescence with 750 nm light excitation. DiR-labeled MSCs further exhibited PA signals under the application of a 680–980 nm laser pulse, allowing the tracking of their localization in heart tissues through both fluorescence molecular tomography (FMT) and multispectral optoacoustic tomography (MSOT) after being intramyocardially injected ex vivo. FMT-MSOT dual imaging technique-based stem cell tracking was confirmed to provide highly spatio-specific and quantitative 3-dimensional information about the biodistribution of transplanted stem cells.

**Figure 5:**
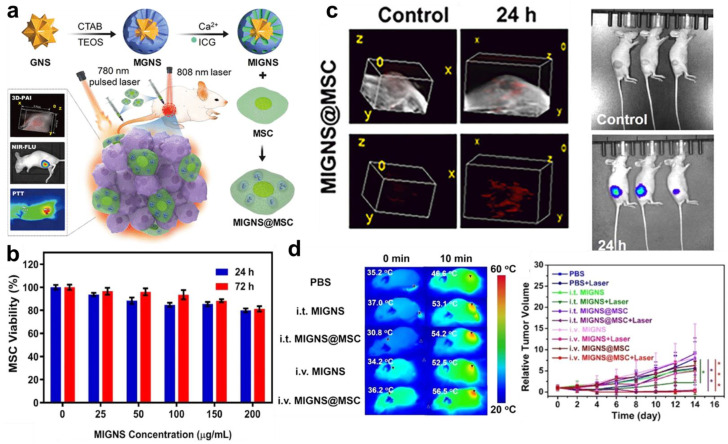
Fluorescence-PA bimodal stem cell labeling. (**a**) A scheme for the brief explanation of ICG-integrated MIGNS-based fluorescence-PA bimodal labeling of MSCs. (**b**) MSC viability after MIGNs treatment. (**c**) Volume-rendered 3D PA images and US images of MSCs in the tumor. Additional fluorescence images of MSCs in the tumor. (**d**) In vivo photothermal effect and tumor growth after laser irradiation by MINGs in MSCs. (*), (**) and (***) indicate difference at the *p* < 0.05, *p* < 0.01 and *p* < 0.001 significance, respectively. Reproduced with permission from [41]. ACS Publications, 2022.

Although organic dyes exhibit fluorescence-PA dual imaging properties by themselves, dye-incorporated nanoparticles are still a good option for stem cell labeling as they can enhance the PA effect and prolong the labeling duration. W. Cai et al. produced NIR-II fluorescent dye (H2)-modified melanin nanoparticles (MNP-PEG-H2) and used them for hUC-MSC labeling and NIR-II fluorescence-PA bimodal tracking [151]. H2 dyes were chemically attached to the surface of pegylated melanin nanoparticles to obtain MNP-PEG-H2s with 23.7 nm of average sizes, wherein H2 dyes emitted fluorescence with 808 nm excitation while melanin nanoparticles generated PA signals under the irradiation of a 680–980 nm laser pulse. Notably, MNP-PEG-H2s were actively taken up by hUC-MSCs and remained inside the hUC-MSCs for a prolonged period without impairing the cell viability or functionality. In the in vivo experiments using acute liver failure mouse models, the migration of MNP-PEG-H2-labeled hUC-MSCs to injured liver tissues could be observed via their long-term tracking with NIR-II fluorescence-PA bimodal imaging. In another study by P. Ning et al., ICG-integrated mesoporous silica-coated gold nanostars (MIGNSs) were designed for fluorescence-PA dual tracking of MSCs (Figure 5a) [41]. ICGs were loaded inside MIGNSs to endow fluorescence emission properties, and MIGNSs exerted strong PA signals with the cooperative effect of ICGs and gold nanostars. MSCs were efficiently labeled with MIGNSs and did not show any serious reduction in their viability (Figure 5b). Through the long-term fluorescence-PA bimodal tracking of MIGNS-labeled MSCs in breast tumor-bearing mice, the homing of intravenously administered MSCs could be monitored with high resolution (Figure 5c). In addition to the bimodal tracking, MIGNSs facilitated the imaging-guided photothermal therapy of breast cancer due to heat generation by ICGs and gold nanostars under 808 nm laser irradiation, demonstrating the theranostic efficacy of MIGNS-labeled MSCs (Figure 5d).

### 3.4. Fluorescence-CT Dual Imaging

CT is a practical bioimaging technique that can produce anatomic images with high resolution using X-ray irradiation. Since the components in the body, including bones, soft tissues, water, and air, have different X-ray absorption properties from one another, detailed structural images of the whole body can be constructed via CT imaging [152]. The administration of CT contrast agents containing elements with high X-ray attenuation coefficients, such as metal nanoparticles or iodinated molecules, is available to highlight the region of interest in CT imaging [42,153,154]. The combination of fluorescence imaging with CT analysis for stem cell tracking is an effective strategy to designate the 3-dimensional location of transplanted stem cells throughout the body with great spatiotemporal precision [155,156]. Moreover, fluorescence–CT bimodal imaging would offset the short tissue penetration depth of fluorescence imaging through the use of X-rays with excellent tissue penetrating ability while overcoming the low sensitivity of CT imaging in soft tissue contrast as well [157,158]. P. D. Nallathamby et al. proposed CY5 dye-loaded gold-silica core-shell nanoparticles (Au@SiO_2_(CY5)) for the immuno-targeted fluorescence–CT bimodal in vivo imaging of cancer stem cells [159]. CD133 antibodies (anti-CD133) were introduced on the surface of Au@SiO_2_(CY5) to target CD133-overexpressing SKOV3-IP cancer cells. The intravenously administered Au@SiO_2_(CY5)-anti-CD133 into tumor-xenograft mice was specifically bound to cancer stem cells and led to their contrast enhancement, which was detectable through fluorescence and CT imaging. Meanwhile, J. Huang, et al. performed the fluorescence–CT dual tracking of implanted MSCs in pulmonary fibrosis-induced mice using gold nanoparticle-based labeling probes [160]. The labeling probes were fabricated through the successive electrostatic adsorption of ICGs and PLL on the surface of albumin-coated gold nanoparticles (AA), obtaining AA@ICG@PLLs with 12.2 nm ± 1.59 nm in size. The zeta potential of AA@ICG@PLLs was measured to be highly positive due to the presence of PLL, which improved their labeling efficiency and durability against MSCs. The incubation of MSCs with AA@ICG@PLLs at a concentration of up to 200 μg/mL endowed them with sufficient fluorescence and X-ray visibility without causing any significant decrease in cell viability, proliferation, or differentiation. Over 7 days of monitoring the behavior of AA@ICG@PLL-labeled MSCs in pulmonary fibrosis models, the migration of MSCs to damaged lung tissues was detected with remarkable spatial resolution via fluorescence–CT dual imaging. Going further, J. S. Park et al. designed fluorescent dye and plasmid DNA (pDNA)-incorporated gold nanoparticles for the application of fluorescence–CT dual imaging-guided stem cell therapy [161]. A total of ~100 nm-sized gold nanoparticles were coated with catechol-functionalized branched PEI (C-bPEI), and RITC dyes were covalently bonded to the C-bPEI coat, forming RITC-labeled C-bPEI-coated gold nanoparticles (M-NTs). Subsequently, pDNAs inducing EGFP expression in hMSCs were physically loaded onto the M-NTs (M-NT/pDNA complexes). hMSCs were sufficiently labeled with M-NT/pDNA complexes due to the positive surface charges of the complexes, bringing about the enhancement in transfection efficiency of co-delivered pDNA as well. High level of EGFP expression was observed in hMSCs after culturing with M-NT/pDNA complexes, wherein neither endocytosis of M-NT/pDNA complexes nor transfection of pDNAs caused any critical cytotoxicity. M-NT/pDNA complex-treated hMSCs were traceable through fluorescence–CT bimodal imaging over 14 days in vivo when they were subcutaneously injected into normal mice. Notably, the exact region where hMSCs were localized could be conveniently identified, validating the complementary performance of fluorescence–CT dual imaging.

### 3.5. Fluorescence-MR Dual Imaging

MR imaging is a highly accurate and precise imaging modality that describes the details of internal structures within the body using a magnetic field and radio waves. MR imaging is conducted by polarizing and exciting protons in the body (mainly in water) under the magnetic and radiofrequency fields and subsequently scanning the radiofrequency signals generated during the spin relaxation of excited protons [162,163,164]. The excited protons exhibit different relaxation times depending on their surrounding molecular environments, which allows for the depiction of detailed images of soft tissues. The relaxation time is divided into T1 and T2 according to the relaxation axis, and different MR images are produced through T1 and T2 measurements. MR contrast agents can be additionally applied to emphasize the target tissues, wherein contrast agents for T1- and T2-weighted imaging are distinguished from each other [165,166]. There are an exceptionally large number of experimental cases of fluorescence–MR dual imaging-based stem cell tracking compared to other bimodal imaging methods, mainly attributed to its outstanding advantages. First, similar to CT imaging, MR imaging can produce high-resolution anatomic mapping that cannot be acquired through molecular imaging modalities, including fluorescence imaging. In addition, MR imaging is usually safer than CT as it does not require X-ray irradiation, and it presents better resolution in soft tissues, which is especially beneficial in revealing the microstructure of tissues [167,168,169]. Therefore, fluorescence–MR dual imaging is considered a powerful modality by which the combination of functional and anatomical information can be implemented to empower the reliability of in vivo stem cell tracking analysis. For fluorescence–MR bimodal tracking of hMSCs, mesoporous silica nanoparticles containing both gadolinium ion(Gd^3+^)-based MR contrast agents and fluorescent dyes were investigated [170]. Gd^3+^-chelated small molecules are the most typical MR contrast agent for T1-weighted imaging; some of them are already clinically available, such as Gadavist^®^, Ablavar^®^, and Eovist^®^ [171,172,173]. The Gd^3+^-chelated diethylenetriaminepentaacetic acid (DTPA) ligands and FITC dyes were chemically conjugated onto mesoporous silica nanoparticles to synthesize Gd-Dye@MSNs, and the synthesized Gd-Dye@MSNs were taken up by hMSCs for their labeling. Gd-Dye@MSN-labeled hMSCs exerted sufficient visibility in both fluorescence and T1-weighted MR imaging in vitro, and their viability, proliferation, and differentiation properties were not diminished after labeling. The in vivo MR imaging of the mouse brain was carried out after the injection of Gd-Dye@MSN-labeled hMSCs into brain tissues, showing the consistent highlight of the injected regions for 14 days. The fluorescence-T1-weighted MR bimodal stem cell labeling was also examined elsewhere, wherein Gd^3+^-functionalized fluorescence carbon dots (Gd-CDs) were employed as dual labeling probes [174]. Gd-CDs with average sizes of 2.9 nm were prepared via a one-step hydrothermal method with GdCl_3_ salts, citrate acids, and ethylene diamines, and the obtained Gd-CDs emitted fluorescence at 450 nm with 350 nm light excitation. The Gd^3+^ content in Gd-CDs was measured to be 18.2% (*w*/*w*), which induced bright contrast in T1-weighted MR imaging. hMSCs were labeled with Gd-CDs through endocytosis, and no cytotoxicity or cell proliferation disturbance due to toxic Gd^3+^ release was observed at a Gd-CD concentration of up to 200 μM, suggesting a promising strategy for fluorescence–MR dual stem cell tracking. Apart from Gd^3+^-based probes, manganese ions (Mn^2+^) or oxides (MnOxs) were known for their T1-weighted MR contrast properties as well, but not many studies on their application in fluorescence-T1-weighted MR dual labeling of stem cells have been reported yet [175,176].

In most clinical or research cases, contrast-enhanced T2-weighted MR imaging is performed using superparamagnetic iron oxide nanoparticles (SPIONs) as MR contrast agents due to their remarkable biocompatibility, clinical availability, and convenience in their modification [177,178]. J.-B. Qin et al. labeled GFP-expressing adipose-derived stem cells (GFP-ADSCs) with SPIONs for their in vivo tracking via fluorescence-T2-weighted MR dual imaging [179]. The SPION labeling of GFP-ADSCs promoted the long-term monitoring of transplanted GFP-ADSCs in the injured carotid artery for 30 days through fluorescence and T2-weighted MR imaging. In the study by Y. Wang et al., fluorescence dye-conjugated SPIONs were investigated for fluorescence-T2-weighted dual imaging of stem cells [180]. SPIONs were coated with silica and subsequently conjugated with rhodamine Bs to produce fluorescent magnetite nanoclusters (FMNCs), and MSCs were labeled with FMNCs for their detection in vivo. When FMNC-labeled MSCs were injected into the brain tissues of normal mice, they were trackable via fluorescence and MR imaging over 30 days. Moreover, the localization of FMNC-labeled MSCs in occluded middle cerebral arteries was detectable through MR analysis. Other researchers have also developed various types of fluorescence dye-functionalized SPIONs, such as Rhodamin B-conjugated SPIONs, FITC-conjugated polymer/SPION nanocomposites, and SPION and IR-780 dye-containing self-assembled polymeric nanoparticles, demonstrating their effectiveness in fluorescence-T2-weighted dual labeling [181,182,183,184]. Further, W. Park et al. developed multimodal transfection agents (MTAs) by successively coating SPIONs with catechol-functionalized polymers and RITC-conjugated PEIs and exploiting them for simultaneous fluorescence–MR bimodal stem cell labeling and gene delivery [46]. EGFP-expressing plasmids (pEGFPs) were loaded onto MTAs through electrostatic interactions, and the efficiencies of MTAs in cell labeling and intracellular gene transfection were evaluated against hMSCs. MTAs were actively taken up by hMSCs with 70.85% endocytosis efficiency 12 h after the incubation and successfully induced EGFP expression inside the cells at 22.25% transfection efficiency. The transfection efficiency of MTAs was 1.6–1.8-fold higher than that of liposomal pEGFPs or pEGFP/PEI complexes. In the in vivo MR and optical imaging assays, the subcutaneously transplanted hMSCs could be observed over 14 days, signifying the prolonged effect of MTA labeling.

**Figure 6:**
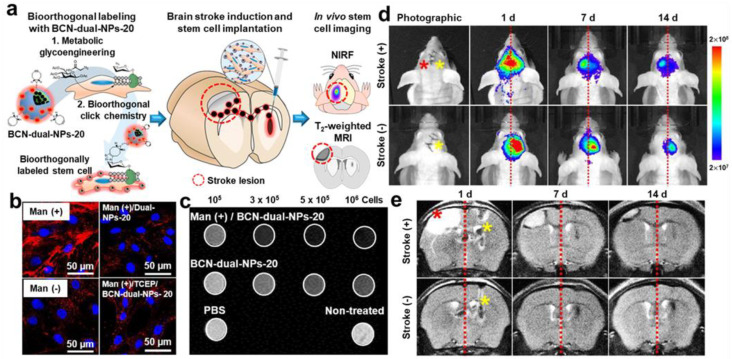
Fluorescence-MR bimodal stem cell labeling. (**a**) Graphical illustration depicts the MSC labeling with fluorescence-MR dual probes via glycoengineering-involved bioorthogonal chemistry and dual imaging-based in vivo tracking of labeled MSCs. (**b**) In vitro confocal fluorescence images of BCN-dual-NP-labeled MSCs with or without the preceding incubation of Ac4Man-NAz. (**c**) In vitro T2-weighted MR phantom images of BCN-dual-NP-labeled MSCs. In vivo (**d**) fluorescence and (**e**) T2-weighted MR images of brain stroke-induced mouse models after transplanting Ac4Man-NAz/BCN-dual-NP-labeled MSCs into brain tissues, visualizing their migration to lesional tissues (red asterisk = stroke-induced region, yellow asterisk = labeled MSC-transplanted site). Reproduced with permission from [43]. ACS Publications, 2019.

Through the introduction of cell-targeting moieties to fluorescence–MR dual labeling probes, their labeling efficiency can be considerably enhanced. X. Xie et al. developed Tat peptide-conjugated paramagnetic UCNPs as fluorescence–MR dual labeling probes for MSC tracking [185]. NaYF4: 20% Yb, 2% Er, and 30% Mn UCNPs were synthesized through the Turkevich–Frens method, which expressed not only fluorescence signals at the 660 nm wavelength but also T1-weighted MR contrast due to the Mn content. The UCNPs were subsequently surface-modified with Tat peptides to form Pep/UCNPs, and the cell labeling performance of Pep/UCNPs against bone marrow-derived MSCs (BMSCs) was compared to that of silica- or DNA-coated ones (SiO_2_/UCNPs or DNA/UCNPs). Among three UCNPs with different coatings, Pep/UCNPs were labeled to BMSCs at the most immediate rate and with the greatest amounts while showing the lowest cytotoxicity. Pep/UCNP-labeled BMSCs were intravenously injected into normal mice, whose biodistribution for 24 h was clearly visible via in vivo fluorescence and T1-weighted MR imaging. In another study by S. Lim et al., hMSCs were labeled with fluorescence–MR dual imaging probes through metabolic glycoengineering-involved bioorthogonal chemistry (Figure 6a) [43]. For the preparation of dual imaging probes, SPIONs were encapsulated in BCN-conjugated glycol chitosan nanoparticles, and Cy5.5 dyes were subsequently conjugated onto the nanoparticles to obtain BCN-dual-NPs. hMSCs were successively incubated with Ac_4_ManNAz and BCN-dual-NPs for labeling, which showed a far higher labeling efficiency of 98.7% than only BCN-dual-NP-treated ones (13.3%) after 6 h incubation (Figure 6b). The T2-weighted MR contrast of Ac_4_ManNAz/BCN-dual-NP-labeled hMSCs was confirmed through the in vitro phantom test (Figure 6c). When Ac_4_ManNAz/BCN-dual-NP-labeled hMSCs were implanted into the brain tissues of photothrombotic stroke-induced mice, their gradual migration to the lesional area was detectable through both fluorescence and MR imaging for 14 days, indicating the desirable stem cell labeling ability of BCN-dual-NPs with Ac_4_ManNAz treatment (Figure 6c,d).

In addition to fluorescence–MR bimodal imaging, several studies have reported the results of triple imaging modality-based stem cell labeling and tracking. NaYF_4_:Yb and Tm@NaGdF_4_ core/shell upconversion nanocrystals (UCNs) were reported as fluorescence–CT–MR trimodal labeling probes for stem cell tracking [186]. UCNs elicited high T1-weighted contrast in MR imaging due to the Gd content in their shells, and the UCN-composing elements with high atomic numbers capacitated their visualization through CT as well. After the complexation with commercial transfecting agents to prepare UCN-TSs, the UCN-TSs were endocytosed into BMSCs for trimodal labeling, which did not affect the viability or differentiation capability of BMScs at a UCN-TS concentration of up to 250 μg/mL. In the in vivo cell transplantation and tracking analysis, the accumulation of UCN-TS-labeled BMSCs in bone defect regions was detectable via fluorescence, CT, and MR imaging upon their injection into rabbit models. The in vivo stem cell tracking using a fluorescence–MR–single photon emission computed tomography (SPECT) trimodal labeling probe was also conducted by Y. Tang et al. [187]. The trimodal probe was produced by conjugating fluorescent dyes and ^125^I radioisotopes consecutively onto the silica-coated SPIONs, resulting in ^125^IfSiO4@SPIOs. ^125^IfSiO4@SPIOs promoted the observation of labeled MSCs through fluorescence, T2-weighted MR, and SPECT imaging without causing significant cytotoxicity, even under weak radiation. Notably, ^125^IfSiO4@SPIO-labeled MSCs were trackable over 14 days through MR and SPECT imaging when they were intravenously or intracranially transplanted to ischemic rat models, wherein the trimodal imaging provided exclusively high accuracy in their tracking.

## 4. Conclusions

Due to their ability to transform into diverse morphologies, stem cell treatment offers superior therapeutic effects for a wide range of diseases. However, the multiple transformation possibilities pose significant risks, especially if the stem cell migrates to an unwanted site with an uncontrollable proliferation rate. Therefore, tracking stem cells is crucial for safe and effective treatment. Fluorescence imaging emerges as the ideal choice for tracking treated stem cells with the advantages of its high resolution, real-time monitoring, and multi-fluorescence detection capabilities. Nevertheless, despite the benefits of fluorescence imaging, its limitations necessitate combined multimodal imaging approaches that leverage the strengths of MRI, PET, and other techniques. These combination strategies effectively address the vulnerabilities of single fluorescence imaging, ensuring a safe and precise diagnosis. By harnessing the capabilities of these advanced imaging and labeling techniques, we are moving closer to fully realizing the therapeutic potential of stem cells, ensuring their safe and effective application in the dynamic field of regenerative medicine. 

## References

[1] Yin J.Q., Zhu J., Ankrum J.A. (2019). Manufacturing of primed mesenchymal stromal cells for therapy. Nat. Biomed. Eng..

[2] Ancans J. (2012). Cell therapy medicinal product regulatory framework in Europe and its application for MSC-based therapy development. Front. Immunol..

[3] Hoang D.M., Pham P.T., Bach T.Q., Ngo A.T.L., Nguyen Q.T., Phan T.T.K., Nguyen G.H., Le P.T.T., Hoang V.T., Forsyth N.R. (2022). Stem cell-based therapy for human diseases. Signal Transduct. Target. Ther..

[4] Andrzejewska A., Dabrowska S., Lukomska B., Janowski M. (2021). Mesenchymal Stem Cells for Neurological Disorders. Adv. Sci..

[5] Datta I., Bhonde R. (2012). Can mesenchymal stem cells reduce vulnerability of dopaminergic neurons in the substantia nigra to oxidative insult in individuals at risk to Parkinson’s disease?. Cell Biol. Int..

[6] Kraus K.H., Kirker-Head C. (2006). Mesenchymal stem cells and bone regeneration. Vet. Surg..

[7] Kasai-Brunswick T.H., Carvalho A.B., Campos de Carvalho A.C. (2021). Stem cell therapies in cardiac diseases: Current status and future possibilities. World J. Stem. Cells.

[8] Yamazaki K., Kawabori M., Seki T., Houkin K. (2020). Clinical Trials of Stem Cell Treatment for Spinal Cord Injury. Int. J. Mol. Sci..

[9] Kawabori M., Shichinohe H., Kuroda S., Houkin K. (2020). Clinical Trials of Stem Cell Therapy for Cerebral Ischemic Stroke. Int. J. Mol. Sci..

[10] Trounson A., Thakar R.G., Lomax G., Gibbons D. (2011). Clinical trials for stem cell therapies. BMC Med..

[11] Tran C., Damaser M.S. (2015). Stem cells as drug delivery methods: Application of stem cell secretome for regeneration. Adv. Drug Deliv. Rev..

[12] Han X., Alu A., Liu H., Shi Y., Wei X., Cai L., Wei Y. (2022). Biomaterial-assisted biotherapy: A brief review of biomaterials used in drug delivery, vaccine development, gene therapy, and stem cell therapy. Bioact. Mater..

[13] Wu H.-H., Zhou Y., Tabata Y., Gao J.-Q. (2019). Mesenchymal stem cell-based drug delivery strategy: From cells to biomimetic. J. Control. Release.

[14] Villa C., Erratico S., Razini P., Fiori F., Rustichelli F., Torrente Y., Belicchi M. (2010). Stem cell tracking by nanotechnologies. Int. J. Mol. Sci..

[15] Accomasso L., Gallina C., Turinetto V., Giachino C. (2016). Stem Cell Tracking with Nanoparticles for Regenerative Medicine Purposes: An Overview. Stem Cells Int..

[16] Cromer Berman S.M., Walczak P., Bulte J.W. (2011). Tracking stem cells using magnetic nanoparticles. Wiley Interdiscip. Rev. Nanomed. Nanobiotechnol..

[17] Goldring C.E., Duffy P.A., Benvenisty N., Andrews P.W., Ben-David U., Eakins R., French N., Hanley N.A., Kelly L., Kitteringham N.R. (2011). Assessing the safety of stem cell therapeutics. Cell Stem. Cell.

[18] Barberini D.J., Aleman M., Aristizabal F., Spriet M., Clark K.C., Walker N.J., Galuppo L.D., Amorim R.M., Woolard K.D., Borjesson D.L. (2018). Safety and tracking of intrathecal allogeneic mesenchymal stem cell transplantation in healthy and diseased horses. Stem Cell Res. Ther..

[19] Cunningham J.J., Ulbright T.M., Pera M.F., Looijenga L.H. (2012). Lessons from human teratomas to guide development of safe stem cell therapies. Nat. Biotechnol..

[20] Sharpe M.E., Morton D., Rossi A. (2012). Nonclinical safety strategies for stem cell therapies. Toxicol. Appl. Pharmacol..

[21] Gordeeva O.F. (2011). Pluripotent cells in embryogenesis and in teratoma formation. J. Stem Cells.

[22] Nguyen P.K., Nag D., Wu J.C. (2010). Methods to assess stem cell lineage, fate and function. Adv. Drug Deliv. Rev..

[23] Xu C., Mu L., Roes I., Miranda-Nieves D., Nahrendorf M., Ankrum J.A., Zhao W., Karp J.M. (2011). Nanoparticle-based monitoring of cell therapy. Nanotechnology.

[24] Chirieleison S.M., Bissell T.A., Scelfo C.C., Anderson J.E., Li Y., Koebler D.J., Deasy B.M. (2011). Automated live cell imaging systems reveal dynamic cell behavior. Biotechnol. Prog..

[25] Song J.H., Lee S.M., Yoo K.H. (2018). Label-free and real-time monitoring of human mesenchymal stem cell differentiation in 2D and 3D cell culture systems using impedance cell sensors. RSC Adv..

[26] Lee S., Kim M.S., Patel K.D., Choi H., Thangam R., Yoon J., Koo T.M., Jung H.J., Min S., Bae G. (2021). Magnetic Control and Real-Time Monitoring of Stem Cell Differentiation by the Ligand Nanoassembly. Small.

[27] Gamal W., Wu H., Underwood I., Jia J., Smith S., Bagnaninchi P.O. (2018). Impedance-based cellular assays for regenerative medicine. Philos. Trans. R. Soc. Lond. B Biol. Sci..

[28] Ding D., Mao D., Li K., Wang X., Qin W., Liu R., Chiam D.S., Tomczak N., Yang Z., Tang B.Z. (2014). Precise and long-term tracking of adipose-derived stem cells and their regenerative capacity via superb bright and stable organic nanodots. ACS Nano.

[29] Duan Q.J., Zhao Z.Y., Zhang Y.J., Fu L., Yuan Y.Y., Du J.Z., Wang J. (2023). Activatable fluorescent probes for real-time imaging-guided tumor therapy. Adv. Drug Deliv. Rev..

[30] Woo Y., Chaurasiya S., O’Leary M., Han E., Fong Y. (2021). Fluorescent imaging for cancer therapy and cancer gene therapy. Mol. Ther. Oncolytics.

[31] Chen J., Dalal R.V., Petrov A.N., Tsai A., O’Leary S.E., Chapin K., Cheng J., Ewan M., Hsiung P.L., Lundquist P. (2014). High-throughput platform for real-time monitoring of biological processes by multicolor single-molecule fluorescence. Proc. Natl. Acad. Sci. USA.

[32] Gioux S., Choi H.S., Frangioni J.V. (2010). Image-guided surgery using invisible near-infrared light: Fundamentals of clinical translation. Mol. Imaging.

[33] Hilderbrand S.A., Weissleder R. (2010). Near-infrared fluorescence: Application to in vivo molecular imaging. Curr. Opin. Chem. Biol..

[34] Kricka L.J., Fortina P. (2009). Analytical ancestry: “firsts” in fluorescent labeling of nucleosides, nucleotides, and nucleic acids. Clin. Chem..

[35] Javois L.C. (1999). Direct immunofluorescent labeling of cells. Methods Mol. Biol..

[36] Rizzo S., Petrella F., Politi L.S., Wang P. (2017). Molecular Imaging of Stems Cells: In Vivo Tracking and Clinical Translation. Stem Cells Int..

[37] Roberts B., Hendershott M.C., Arakaki J., Gerbin K.A., Malik H., Nelson A., Gehring J., Hookway C., Ludmann S.A., Yang R. (2019). Fluorescent Gene Tagging of Transcriptionally Silent Genes in hiPSCs. Stem Cell Rep..

[38] Scandella V., Paolicelli R.C., Knobloch M. (2020). A novel protocol to detect green fluorescent protein in unfixed, snap-frozen tissue. Sci. Rep..

[39] Roberts B., Haupt A., Tucker A., Grancharova T., Arakaki J., Fuqua M.A., Nelson A., Hookway C., Ludmann S.A., Mueller I.A. (2017). Systematic gene tagging using CRISPR/Cas9 in human stem cells to illuminate cell organization. Mol. Biol. Cell.

[40] Haupt A., Grancharova T., Arakaki J., Fuqua M.A., Roberts B., Gunawardane R.N. (2018). Endogenous Protein Tagging in Human Induced Pluripotent Stem Cells Using CRISPR/Cas9. J. Vis. Exp..

[41] Ning P., Chen Y., Bai Q., Xu C., Deng C., Cheng Q., Cheng Y. (2022). Multimodal Imaging-Guided Spatiotemporal Tracking of Photosensitive Stem Cells for Breast Cancer Treatment. ACS Appl. Mater. Interfaces.

[42] Lee N., Choi S.H., Hyeon T. (2013). Nano-sized CT contrast agents. Adv. Mater..

[43] Lim S., Yoon H.Y., Jang H.J., Song S., Kim W., Park J., Lee K.E., Jeon S., Lee S., Lim D.-K. (2019). Dual-modal imaging-guided precise tracking of bioorthogonally labeled mesenchymal stem cells in mouse brain stroke. ACS Nano.

[44] Gigan S., Katz O., de Aguiar H.B., Andresen E.R., Aubry A., Bertolotti J., Bossy E., Bouchet D., Brake J., Brasselet S. (2022). Roadmap on wavefront shaping and deep imaging in complex media. J. Phys.-Photonics.

[45] Ji N. (2017). Adaptive optical fluorescence microscopy. Nat. Methods.

[46] Park W., Yang H.N., Ling D., Yim H., Kim K.S., Hyeon T., Na K., Park K.H. (2014). Multi-modal transfection agent based on monodisperse magnetic nanoparticles for stem cell gene delivery and tracking. Biomaterials.

[47] Gaedicke S., Braun F., Prasad S., Machein M., Firat E., Hettich M., Gudihal R., Zhu X., Klingner K., Schuler J. (2014). Noninvasive positron emission tomography and fluorescence imaging of CD133+ tumor stem cells. Proc. Natl. Acad. Sci. USA.

[48] Kim M.H., Lee Y.J., Kang J.H. (2016). Stem Cell Monitoring with a Direct or Indirect Labeling Method. Nucl. Med. Mol. Imaging.

[49] Lim S., Kim W., Song S., Shim M.K., Yoon H.Y., Kim B.-S., Kwon I.C., Kim K. (2021). Intracellular uptake mechanism of bioorthogonally conjugated nanoparticles on metabolically engineered mesenchymal stem cells. Bioconjugate Chem..

[50] Hsu T.C., Liu K.K., Chang H.C., Hwang E., Chao J.I. (2014). Labeling of neuronal differentiation and neuron cells with biocompatible fluorescent nanodiamonds. Sci. Rep..

[51] Yamamoto N., Tsuchiya H., Hoffman R.M. (2011). Tumor imaging with multicolor fluorescent protein expression. Int. J. Clin. Oncol..

[52] Cubitt A.B., Heim R., Adams S.R., Boyd A.E., Gross L.A., Tsien R.Y. (1995). Understanding, improving and using green fluorescent proteins. Trends Biochem. Sci..

[53] Kamiyama D., Sekine S., Barsi-Rhyne B., Hu J., Chen B., Gilbert L.A., Ishikawa H., Leonetti M.D., Marshall W.F., Weissman J.S. (2016). Versatile protein tagging in cells with split fluorescent protein. Nat. Commun..

[54] Cabantous S., Terwilliger T.C., Waldo G.S. (2005). Protein tagging and detection with engineered self-assembling fragments of green fluorescent protein. Nat. Biotechnol..

[55] Shaner N.C., Patterson G.H., Davidson M.W. (2007). Advances in fluorescent protein technology. J. Cell Sci..

[56] Tsien R.Y. (1998). The green fluorescent protein. Annu. Rev. Biochem..

[57] Remington S.J. (2011). Green fluorescent protein: A perspective. Protein Sci..

[58] Chudakov D.M., Matz M.V., Lukyanov S., Lukyanov K.A. (2010). Fluorescent proteins and their applications in imaging living cells and tissues. Physiol. Rev..

[59] Tao W., Evans B.-G., Yao J., Cooper S., Cornetta K., Ballas C.B., Hangoc G., Broxmeyer H.E. (2007). Enhanced green fluorescent protein is a nearly ideal long-term expression tracer for hematopoietic stem cells, whereas DsRed-express fluorescent protein is not. Stem Cells.

[60] Shichinohe H., Kuroda S., Lee J.-B., Nishimura G., Yano S., Seki T., Ikeda J., Tamura M., Iwasaki Y. (2004). In vivo tracking of bone marrow stromal cells transplanted into mice cerebral infarct by fluorescence optical imaging. Brain Res. Protoc..

[61] Hadjantonakis A.-K., Papaioannou V.E. (2004). Dynamic in vivo imaging and cell tracking using a histone fluorescent protein fusion in mice. BMC Biotechnol..

[62] Torres-Acosta M.A., dos Santos N.V., Ventura S.P., Coutinho J.A., Rito-Palomares M., Pereira J.F. (2021). Economic analysis of the production and recovery of green fluorescent protein using ATPS-based bioprocesses. Sep. Purif. Technol..

[63] Boddington S., Henning T.D., Sutton E.J., Daldrup-Link H.E. (2008). Labeling stem cells with fluorescent dyes for non-invasive detection with optical imaging. JoVE.

[64] Boddington S.E., Sutton E.J., Henning T.D., Nedopil A.J., Sennino B., Kim A., Daldrup-Link H.E. (2011). Labeling human mesenchymal stem cells with fluorescent contrast agents: The biological impact. Mol. Imaging Biol..

[65] Andrzejewska A., Jablonska A., Seta M., Dabrowska S., Walczak P., Janowski M., Lukomska B. (2019). Labeling of human mesenchymal stem cells with different classes of vital stains: Robustness and toxicity. Stem Cell Res. Ther..

[66] Lin C.-S., Xin Z.-C., Dai J., Lue T.F. (2013). Commonly used mesenchymal stem cell markers and tracking labels: Limitations and challenges. Histol. Histopathol..

[67] Cheng C., Trzcinski O., Doering L.C. (2014). Fluorescent labeling of dendritic spines in cell cultures with the carbocyanine dye “DiI”. Front. Neuroanat..

[68] Mohtasebi M.S., Nasri F., Kamali Sarvestani E. (2014). Effect of DiD carbocyanine dye labeling on immunoregulatory function and differentiation of mice mesenchymal stem cells. Stem Cells Int..

[69] Schultz M., Müller R., Ermakova Y., Hoffmann J.E., Schultz C. (2022). Membrane-Permeant, Bioactivatable Coumarin Derivatives for In-Cell Labelling. ChemBioChem.

[70] Froelich K., Steussloff G., Schmidt K., Ramos Tirado M., Technau A., Scherzed A., Hackenberg S., Radeloff A., Hagen R., Kleinsasser N. (2013). DiI labeling of human adipose-derived stem cells: Evaluation of DNA damage, toxicity and functional impairment. Cells Tissues Organs.

[71] Odintsov B., Chun J.L., Berry S.E., Turksen K. (2013). Whole body MRI and fluorescent microscopy for detection of stem cells labeled with superparamagnetic iron oxide (SPIO) nanoparticles and DiI following intramuscular and systemic delivery. Imaging and Tracking Stem Cells. Methods in Molecular Biology.

[72] Nagyova M., Slovinska L., Blasko J., Grulova I., Kuricova M., Cigankova V., Harvanova D., Cizkova D. (2014). A comparative study of PKH67, DiI, and BrdU labeling techniques for tracing rat mesenchymal stem cells. Vitr. Cell. Dev. Biol. Anim..

[73] Ji F., Duan H.-G., Zheng C.-Q., Li J. (2015). Comparison of chloromethyl-dialkylcarbocyanine and green fluorescent protein for labeling human umbilical mesenchymal stem cells. Biotechnol. Lett..

[74] Li M., Luo X., Lv X., Liu V., Zhao G., Zhang X., Cao W., Wang R., Wang W. (2016). In vivo human adipose-derived mesenchymal stem cell tracking after intra-articular delivery in a rat osteoarthritis model. Stem Cell Res. Ther..

[75] Chen J., Li D., Li H., Zhu K., Shi L., Fu X. (2023). Cell membrane-targeting NIR fluorescent probes with large Stokes shifts for ultralong-term transplanted neural stem cell tracking. Front. Bioeng. Biotechnol..

[76] Weir C., Morel-Kopp M.-C., Gill A., Tinworth K., Ladd L., Hunyor S.N., Ward C. (2008). Mesenchymal stem cells: Isolation, characterisation and in vivo fluorescent dye tracking. Heart Lung Circ..

[77] Sabapathy V., Mentam J., Jacob P.M., Kumar S. (2015). Noninvasive optical imaging and in vivo cell tracking of indocyanine green labeled human stem cells transplanted at superficial or in-depth tissue of SCID mice. Stem Cells Int..

[78] Zhang C., Tan X., Tan L., Liu T., Liu D., Zhang L., Fan S., Su Y., Cheng T., Zhou Y. (2011). Labeling stem cells with a near-infrared fluorescent heptamethine dye for noninvasive optical tracking. Cell Transplant..

[79] Zhang X., Bloch S., Akers W., Achilefu S. (2012). Near-infrared molecular probes for in vivo imaging. Curr. Protoc. Cytom..

[80] Ruedas-Rama M.J., Walters J.D., Orte A., Hall E.A. (2012). Fluorescent nanoparticles for intracellular sensing: A review. Anal. Chim. Acta.

[81] Wang Y., Xu C., Ow H. (2013). Commercial nanoparticles for stem cell labeling and tracking. Theranostics.

[82] Mogharbel B.F., Francisco J.C., Irioda A.C., Dziedzic D.S.M., Ferreira P.E., De Souza D., De Souza C.M.C.O., Neto N.B., Guarita-Souza L.C., Franco C.R.C. (2018). Fluorescence properties of curcumin-loaded nanoparticles for cell tracking. Int. J. Nanomed..

[83] Bao H., Li Y., Yu C., Li X., Wang Y., Gao L., Huang J., Zhang Z. (2022). DNA-coated gold nanoparticles for tracking hepatocyte growth factor secreted by transplanted mesenchymal stem cells in pulmonary fibrosis therapy. Biomater. Sci..

[84] Cova L., Bigini P., Diana V., Sitia L., Ferrari R., Pesce R.M., Khalaf R., Bossolasco P., Ubezio P., Lupi M. (2013). Biocompatible fluorescent nanoparticles for in vivo stem cell tracking. Nanotechnology.

[85] Liu S., Tay L.M., Anggara R., Chuah Y.J., Kang Y. (2016). Long-term tracking mesenchymal stem cell differentiation with photostable fluorescent nanoparticles. ACS Appl. Mater. Interfaces.

[86] Yeo D., Wiraja C., Chuah Y.J., Gao Y., Xu C. (2015). A nanoparticle-based sensor platform for cell tracking and status/function assessment. Sci. Rep..

[87] Chung T.-H., Wu S.-H., Yao M., Lu C.-W., Lin Y.-S., Hung Y., Mou C.-Y., Chen Y.-C., Huang D.-M. (2007). The effect of surface charge on the uptake and biological function of mesoporous silica nanoparticles in 3T3-L1 cells and human mesenchymal stem cells. Biomaterials.

[88] Gao Y., Wang Y., Fu A., Shi W., Yeo D., Luo K.Q., Ow H., Xu C. (2015). Tracking mesenchymal stem cell tumor-homing using fluorescent silica nanoparticles. J. Mater. Chem. B.

[89] Yang C., Gao S., Song P., Dagnæs-Hansen F., Jakobsen M., Kjems J. (2018). Theranostic niosomes for efficient siRNA/MicroRNA delivery and activatable near-infrared fluorescent tracking of stem cells. ACS Appl. Mater. Interfaces.

[90] Yuan Y., Zhang Z., Hou W., Qin W., Meng Z., Wu C. (2020). In vivo dynamic cell tracking with long-wavelength excitable and near-infrared fluorescent polymer dots. Biomaterials.

[91] Jin G., Li W., Song F., Zhao J., Wang M., Liu Q., Li A., Huang G., Xu F. (2020). Fluorescent conjugated polymer nanovector for in vivo tracking and regulating the fate of stem cells for restoring infarcted myocardium. Acta Biomater..

[92] Lee S., Yoon H.I., Na J.H., Jeon S., Lim S., Koo H., Han S.-S., Kang S.-W., Park S.-J., Moon S.-H. (2017). In vivo stem cell tracking with imageable nanoparticles that bind bioorthogonal chemical receptors on the stem cell surface. Biomaterials.

[93] Yun W.S., Shim M.K., Lim S., Song S., Kim J., Yang S., Hwang H.S., Kim M.R., Yoon H.Y., Lim D.-K. (2022). Mesenchymal stem cell-mediated deep tumor delivery of gold nanorod for photothermal therapy. Nanomaterials.

[94] Kairdolf B.A., Smith A.M., Stokes T.H., Wang M.D., Young A.N., Nie S. (2013). Semiconductor quantum dots for bioimaging and biodiagnostic applications. Annu. Rev. Anal. Chem..

[95] Bilan R., Nabiev I., Sukhanova A. (2016). Quantum dot-based nanotools for bioimaging, diagnostics, and drug delivery. ChemBioChem.

[96] Martynenko I., Litvin A., Purcell-Milton F., Baranov A., Fedorov A., Gun’Ko Y. (2017). Application of semiconductor quantum dots in bioimaging and biosensing. J. Mater. Chem. B.

[97] Wan T., Aleem A.R., Huang S., Chen R., Zhou Z., Sun M., He J., Yu L., Wen H. (2023). Autofluorescence free functionalized upconversion nanoparticles-based turn-on aptasensor for highly sensitive and selective sensing of antibiotics. Mater. Today Adv..

[98] Lin S., Xie X., Patel M.R., Yang Y.-H., Li Z., Cao F., Gheysens O., Zhang Y., Gambhir S.S., Rao J.H. (2007). Quantum dot imaging for embryonic stem cells. BMC Biotechnol..

[99] Chen G., Tian F., Li C., Zhang Y., Weng Z., Zhang Y., Peng R., Wang Q. (2015). In vivo real-time visualization of mesenchymal stem cells tropism for cutaneous regeneration using NIR-II fluorescence imaging. Biomaterials.

[100] Lei Y., Tang H., Yao L., Yu R., Feng M., Zou B. (2008). Applications of mesenchymal stem cells labeled with Tat peptide conjugated quantum dots to cell tracking in mouse body. Bioconjugate Chem..

[101] Li J., Lee W.Y., Wu T., Xu J., Zhang K., Li G., Xia J., Bian L. (2016). Multifunctional quantum dot nanoparticles for effective differentiation and long-term tracking of human mesenchymal stem cells in vitro and in vivo. Adv. Healthc. Mater..

[102] Malina T., Poláková K., Skopalík J., Milotová V., Holá K., Havrdová M., Tománková K.B., Čmiel V., Šefc L., Zbořil R. (2019). Carbon dots for in vivo fluorescence imaging of adipose tissue-derived mesenchymal stromal cells. Carbon.

[103] Zhang M., Bai L., Shang W., Xie W., Ma H., Fu Y., Fang D., Sun H., Fan L., Han M. (2012). Facile synthesis of water-soluble, highly fluorescent graphene quantum dots as a robust biological label for stem cells. J. Mater. Chem..

[104] Yan J., Hou S., Yu Y., Qiao Y., Xiao T., Mei Y., Zhang Z., Wang B., Huang C.-C., Lin C.-H. (2018). The effect of surface charge on the cytotoxicity and uptake of carbon quantum dots in human umbilical cord derived mesenchymal stem cells. Colloids Surf. B Biointerfaces.

[105] Schrand A.M., Hens S.A.C., Shenderova O.A. (2009). Nanodiamond particles: Properties and perspectives for bioapplications. Crit. Rev. Solid. State Mater. Sci..

[106] Alkahtani M.H., Alghannam F., Jiang L., Almethen A., Rampersaud A.A., Brick R., Gomes C.L., Scully M.O., Hemmer P.R. (2018). Fluorescent nanodiamonds: Past, present, and future. Nanophotonics.

[107] Bottrill M., Green M. (2011). Some aspects of quantum dot toxicity. Chem. Commun..

[108] Williams O.A., Nesladek M., Daenen M., Michaelson S., Hoffman A., Osawa E., Haenen K., Jackman R. (2008). Growth, electronic properties and applications of nanodiamond. Diam. Relat. Mater..

[109] Fang C.Y., Vaijayanthimala V., Cheng C.A., Yeh S.H., Chang C.F., Li C.L., Chang H.C. (2011). The exocytosis of fluorescent nanodiamond and its use as a long-term cell tracker. Small.

[110] Hsiao W.W.-W., Hui Y.Y., Tsai P.-C., Chang H.-C. (2016). Fluorescent nanodiamond: A versatile tool for long-term cell tracking, super-resolution imaging, and nanoscale temperature sensing. Acc. Chem. Res..

[111] Wu T.-J., Tzeng Y.-K., Chang W.-W., Cheng C.-A., Kuo Y., Chien C.-H., Chang H.-C., Yu J. (2013). Tracking the engraftment and regenerative capabilities of transplanted lung stem cells using fluorescent nanodiamonds. Nat. Nanotechnol..

[112] Lin H.H., Lee H.W., Lin R.J., Huang C.W., Liao Y.C., Chen Y.T., Fang J.M., Lee T.C., Yu A.L., Chang H.C. (2015). Tracking and finding slow-proliferating/quiescent cancer stem cells with fluorescent nanodiamonds. Small.

[113] Su L.-J., Wu M.-S., Hui Y.Y., Chang B.-M., Pan L., Hsu P.-C., Chen Y.-T., Ho H.-N., Huang Y.-H., Ling T.-Y. (2017). Fluorescent nanodiamonds enable quantitative tracking of human mesenchymal stem cells in miniature pigs. Sci. Rep..

[114] Zhu X., Su Q., Feng W., Li F. (2017). Anti-Stokes shift luminescent materials for bio-applications. Chem. Soc. Rev..

[115] Zhan Q., He S., Qian J., Cheng H., Cai F. (2013). Optimization of optical excitation of upconversion nanoparticles for rapid microscopy and deeper tissue imaging with higher quantum yield. Theranostics.

[116] Tian Z., Chen G., Li X., Liang H., Li Y., Zhang Z., Tian Y. (2010). Autofluorescence-free in vivo multicolor imaging using upconversion fluoride nanocrystals. Lasers Med. Sci..

[117] Zhao L., Kutikov A., Shen J., Duan C., Song J., Han G. (2013). Stem cell labeling using polyethylenimine conjugated (α-NaYbF4: Tm3+)/CaF2 upconversion nanoparticles. Theranostics.

[118] Wang C., Cheng L., Xu H., Liu Z. (2012). Towards whole-body imaging at the single cell level using ultra-sensitive stem cell labeling with oligo-arginine modified upconversion nanoparticles. Biomaterials.

[119] Li J., Lee W.Y.-W., Wu T., Xu J., Zhang K., Wong D.S.H., Li R., Li G., Bian L. (2016). Near-infrared light-triggered release of small molecules for controlled differentiation and long-term tracking of stem cells in vivo using upconversion nanoparticles. Biomaterials.

[120] Li J., Leung C.W.T., Wong D.S.H., Xu J., Li R., Zhao Y., Yung C.Y.Y., Zhao E., Tang B.Z., Bian L. (2017). Photocontrolled SiRNA delivery and biomarker-triggered luminogens of aggregation-induced emission by up-conversion NaYF4: Yb3+ Tm3+@ SiO_2_ nanoparticles for inducing and monitoring stem-cell differentiation. ACS Appl. Mater. Interfaces.

[121] Zhao J., Chen J., Ma S., Liu Q., Huang L., Chen X., Lou K., Wang W. (2018). Recent developments in multimodality fluorescence imaging probes. Acta Pharm. Sin. B.

[122] Lee S.Y., Jeon S.I., Jung S., Chung I.J., Ahn C.-H. (2014). Targeted multimodal imaging modalities. Adv. Drug Deliv. Rev..

[123] Crosignani V., Dvornikov A., Aguilar J.S., Stringari C., Edwards R., Mantulin W.W., Gratton E. (2012). Deep tissue fluorescence imaging and in vivo biological applications. J. Biomed. Opt..

[124] Ghoroghchian P.P., Therien M.J., Hammer D.A. (2009). In vivo fluorescence imaging: A personal perspective. Wiley Interdiscip. Rev. Nanomed. Nanobiotechnol..

[125] Srinivas M., Melero I., Kaempgen E., Figdor C.G., de Vries I.J.M. (2013). Cell tracking using multimodal imaging. Contrast Media Mol. Imaging.

[126] Badr C.E., Tannous B.A. (2011). Bioluminescence imaging: Progress and applications. Trends Biotechnol..

[127] Contag C.H., Bachmann M.H. (2002). Advances in in vivo bioluminescence imaging of gene expression. Annu. Rev. Biomed. Eng..

[128] Rajapakse H.E., Gahlaut N., Mohandessi S., Yu D., Turner J.R., Miller L.W. (2010). Time-resolved luminescence resonance energy transfer imaging of protein–protein interactions in living cells. Proc. Natl. Acad. Sci. USA.

[129] Saito K., Nagai T. (2015). Recent progress in luminescent proteins development. Curr. Opin. Chem. Biol..

[130] Ozawa T., Yoshimura H., Kim S.B. (2013). Advances in fluorescence and bioluminescence imaging. Anal. Chem..

[131] Miao Q., Pu K. (2018). Organic semiconducting agents for deep-tissue molecular imaging: Second near-infrared fluorescence, self-luminescence, and photoacoustics. Adv. Mater..

[132] Huang D., Lin S., Wang Q., Zhang Y., Li C., Ji R., Wang M., Chen G., Wang Q. (2019). An NIR-II Fluorescence/Dual Bioluminescence Multiplexed Imaging for In Vivo Visualizing the Location, Survival, and Differentiation of Transplanted Stem Cells. Adv. Funct. Mater..

[133] Su L.-J., Lin H.-H., Wu M.-S., Pan L., Yadav K., Hsu H.-H., Ling T.-Y., Chen Y.-T., Chang H.-C. (2019). Intracellular delivery of luciferase with fluorescent nanodiamonds for dual-modality imaging of human stem cells. Bioconjugate Chem..

[134] Bar-Shalom R., Valdivia A.Y., Blaufox M.D. (2000). PET Imaging in Oncology.

[135] Basu S., Kwee T.C., Surti S., Akin E.A., Yoo D., Alavi A. (2011). Fundamentals of PET and PET/CT imaging. Ann. N. Y. Acad. Sci..

[136] Ariztia J., Solmont K., Moïse N.P., Specklin S., Heck M.P., Lamande-Langle S., Kuhnast B. (2022). PET/fluorescence imaging: An overview of the chemical strategies to build dual imaging tools. Bioconjugate Chem..

[137] Pham T.T., Lu Z., Davis C., Li C., Sun F., Maher J., Yan R. (2020). Iodine-124 based dual positron emission tomography and fluorescent labeling reagents for in vivo cell tracking. Bioconjugate Chem..

[138] Harmsen S., Medine E.I., Moroz M., Nurili F., Lobo J., Dong Y., Turkekul M., Pillarsetty N.V.K., Ting R., Ponomarev V. (2021). A dual-modal PET/near infrared fluorescent nanotag for long-term immune cell tracking. Biomaterials.

[139] Zettlitz K.A., Tsai W.-T.K., Knowles S.M., Kobayashi N., Donahue T.R., Reiter R.E., Wu A.M. (2018). Dual-Modality Immuno-PET and Near-Infrared Fluorescence Imaging of Pancreatic Cancer Using an Anti–Prostate Stem Cell Antigen Cys-Diabody. J. Nucl. Med..

[140] Yuen R., West F.G., Wuest F. (2022). Dual probes for positron emission tomography (PET) and fluorescence imaging (FI) of cancer. Pharmaceutics.

[141] Attia A.B.E., Balasundaram G., Moothanchery M., Dinish U., Bi R., Ntziachristos V., Olivo M. (2019). A review of clinical photoacoustic imaging: Current and future trends. Photoacoustics.

[142] Upputuri P.K., Das D., Maheshwari M., Yaowen Y., Pramanik M. (2020). Real-time monitoring of temperature using a pulsed laser-diode-based photoacoustic system. Opt. Lett..

[143] Kang J., Zhang H.K., Rahmim A., Wong D.F., Kang J.U., Boctor E.M. (2017). Toward high-speed transcranial photoacoustic imaging using compact near-infrared pulsed LED illumination system. Photons Plus Ultrasound: Imaging and Sensing 2017.

[144] Beard P. (2011). Biomedical photoacoustic imaging. Interface Focus..

[145] Lengenfelder B., Mehari F., Hohmann M., Heinlein M., Chelales E., Waldner M.J., Klämpfl F., Zalevsky Z., Schmidt M. (2019). Remote photoacoustic sensing using speckle-analysis. Sci. Rep..

[146] Sreejith S., Joseph J., Lin M., Menon N.V., Borah P., Ng H.J., Loong Y.X., Kang Y., Yu S.W.-K., Zhao Y. (2015). Near-infrared squaraine dye encapsulated micelles for in vivo fluorescence and photoacoustic bimodal imaging. ACS Nano.

[147] Mokrousov M.D., Thompson W., Ermilov S.A., Abakumova T., Novoselova M.V., Inozemtseva O.A., Zatsepin T.S., Zharov V.P., Galanzha E.I., Gorin D.A. (2021). Indocyanine green dye based bimodal contrast agent tested by photoacoustic/fluorescence tomography setup. Biomed. Opt. Express.

[148] Nguyen V.P., Qian W., Li Y., Liu B., Aaberg M., Henry J., Zhang W., Wang X., Paulus Y.M. (2021). Chain-like gold nanoparticle clusters for multimodal photoacoustic microscopy and optical coherence tomography enhanced molecular imaging. Nat. Commun..

[149] Filippi M., Garello F., Pasquino C., Arena F., Giustetto P., Antico F., Terreno E. (2019). Indocyanine green labeling for optical and photoacoustic imaging of mesenchymal stem cells after in vivo transplantation. J. Biophotonics.

[150] Berninger M.T., Mohajerani P., Wildgruber M., Beziere N., Kimm M.A., Ma X., Haller B., Fleming M.J., Vogt S., Anton M. (2017). Detection of intramyocardially injected DiR-labeled mesenchymal stem cells by optical and optoacoustic tomography. Photoacoustics.

[151] Cai W., Sun J., Sun Y., Zhao X., Guo C., Dong J., Peng X., Zhang R. (2020). NIR-II FL/PA dual-modal imaging long-term tracking of human umbilical cord-derived mesenchymal stem cells labeled with melanin nanoparticles and visible HUMSC-based liver regeneration for acute liver failure. Biomater. Sci..

[152] Kircher M.F., Willmann J.K. (2012). Molecular body imaging: MR imaging, CT, and US. part I. principles. Radiology.

[153] Lusic H., Grinstaff M.W. (2013). X-ray-computed tomography contrast agents. Chem. Rev..

[154] Li J., Chaudhary A., Chmura S.J., Pelizzari C., Rajh T., Wietholt C., Kurtoglu M., Aydogan B. (2010). A novel functional CT contrast agent for molecular imaging of cancer. Phys. Med. Biol..

[155] Zhang L., Yang X.-Q., An J., Zhao S.-D., Zhao T.-Y., Tan F., Cao Y.-C., Zhao Y.-D. (2018). In vivo tumor active cancer targeting and CT-fluorescence dual-modal imaging with nanoprobe based on gold nanorods and InP/ZnS quantum dots. J. Mater. Chem. B.

[156] Xing H., Bu W., Ren Q., Zheng X., Li M., Zhang S., Qu H., Wang Z., Hua Y., Zhao K. (2012). A NaYbF4: Tm3+ nanoprobe for CT and NIR-to-NIR fluorescent bimodal imaging. Biomaterials.

[157] Zhang J., Li C., Zhang X., Huo S., Jin S., An F.-F., Wang X., Xue X., Okeke C., Duan G. (2015). In vivo tumor-targeted dual-modal fluorescence/CT imaging using a nanoprobe co-loaded with an aggregation-induced emission dye and gold nanoparticles. Biomaterials.

[158] Feng J., Chang D., Wang Z., Shen B., Yang J., Jiang Y., Ju S., He N. (2014). A FITC-doped silica coated gold nanocomposite for both in vivo X-ray CT and fluorescence dual modal imaging. Rsc. Adv..

[159] Nallathamby P.D., Dahl K.C., Roeder R.K. In Vivo Detection of Cancer Stem Cells by Dual Mode CT/Fluorescence Using Immunotargeted Nanoparticle Probes. https://abstracts.biomaterials.org/data/papers/2018/abstracts/267.pdf.

[160] Huang J., Ning X., Luo W., Chen M., Wang Z., Zhang W., Zhang Z., Chao J. (2020). CT/NIRF dual-modal imaging tracking and therapeutic efficacy of transplanted mesenchymal stem cells labeled with Au nanoparticles in silica-induced pulmonary fibrosis. J. Mater. Chem. B.

[161] Park J.S., Park W., Kang A.Y., Larson A.C., Kim D.-H., Park K.-H. (2017). Multi-functional nanotracers for image-guided stem cell gene therapy. Nanoscale.

[162] Berger A. (2002). How does it work? Magnetic resonance imaging. BMJ Br. Med. J..

[163] McGowan J.C. (2008). Basic principles of magnetic resonance imaging. Neuroimaging Clin. North. Am..

[164] Van Geuns R.-J.M., Wielopolski P.A., de Bruin H.G., Rensing B.J., Van Ooijen P.M., Hulshoff M., Oudkerk M., de Feyter P.J. (1999). Basic principles of magnetic resonance imaging. Prog. Cardiovasc. Dis..

[165] Xiao Y.-D., Paudel R., Liu J., Ma C., Zhang Z.-S., Zhou S.-K. (2016). MRI contrast agents: Classification and application. Int. J. Mol. Med..

[166] Strijkers G.J., M Mulder W.J., F van Tilborg G.A., Nicolay K. (2007). MRI contrast agents: Current status and future perspectives. Anti-Cancer Agents Med. Chem..

[167] van Beek E.J., Hoffman E.A. (2008). Functional imaging: CT and MRI. Clin. Chest Med..

[168] Chang A.E., Matory Y.L., Dwyer A.J., Hill S.C., Girton M.E., Steinberg S.M., Knop R.H., Frank J.A., Hyams D., Doppman J.L. (1987). Magnetic resonance imaging versus computed tomography in the evaluation of soft tissue tumors of the extremities. Ann. Surg..

[169] Semelka R.C., Armao D.M., Elias J., Huda W. (2007). Imaging strategies to reduce the risk of radiation in CT studies, including selective substitution with MRI. J. Magn. Reson. Imaging Off. J. Int. Soc. Magn. Reson. Med..

[170] Hsiao J.K., Tsai C.P., Chung T.H., Hung Y., Yao M., Liu H.M., Mou C.Y., Yang C.S., Chen Y.C., Huang D.M. (2008). Mesoporous silica nanoparticles as a delivery system of gadolinium for effective human stem cell tracking. Small.

[171] Scott L.J. (2018). Gadobutrol: A review in contrast-enhanced MRI and MRA. Clin. Drug Investig..

[172] Pressacco J., Papas K. (2012). Gadofosveset-enhanced magnetic resonance angiography as a means of evaluating pulmonary arteriovenous malformation: A case report. Magn. Reson. Imaging.

[173] Marks A.L., Hecht S., Stokes J.E., Conklin G.A., Deanna K.H. (2014). Effects of Gadoxetate disodium (Eovist®) contrast on magnetic resonance imaging characteristics of the liver in clinically healthy dogs. Vet. Radiol. Ultrasound.

[174] Chen H., Wang L., Fu H., Wang Z., Xie Y., Zhang Z., Tang Y. (2016). Gadolinium functionalized carbon dots for fluorescence/magnetic resonance dual-modality imaging of mesenchymal stem cells. J. Mater. Chem. B.

[175] Das B., Girigoswami A., Pal P., Dhara S. (2019). Manganese oxide-carbon quantum dots nano-composites for fluorescence/magnetic resonance (T1) dual mode bioimaging, long term cell tracking, and ros scavenging. Mater. Sci. Eng. C.

[176] Chetty S.S., Praneetha S., Vadivel Murugan A., Govarthanan K., Verma R.S. (2019). Human umbilical cord wharton’s jelly-derived mesenchymal stem cells labeled with Mn^2+^ and Gd^3+^ Co-doped CuInS2–ZnS nanocrystals for multimodality imaging in a tumor mice model. ACS Appl. Mater. Interfaces.

[177] Neuwelt A., Sidhu N., Hu C.-A.A., Mlady G., Eberhardt S.C., Sillerud L.O. (2015). Iron-based superparamagnetic nanoparticle contrast agents for MRI of infection and inflammation. AJR. Am. J. Roentgenol..

[178] Antonelli A., Magnani M. (2022). SPIO nanoparticles and magnetic erythrocytes as contrast agents for biomedical and diagnostic applications. J. Magn. Magn. Mater..

[179] Qin J.-B., Li K.-A., Li X.-X., Xie Q.-S., Lin J.-Y., Ye K.-C., Jiang M.-E., Zhang G.-X., Lu X.-W. (2012). Long-term MRI tracking of dual-labeled adipose-derived stem cells homing into mouse carotid artery injury. Int. J. Nanomed..

[180] Wang Y., Xu F., Zhang C., Lei D., Tang Y., Xu H., Zhang Z., Lu H., Du X., Yang G.-Y. (2011). High MR sensitive fluorescent magnetite nanocluster for stem cell tracking in ischemic mouse brain. Nanomed. Nanotechnol. Biol. Med..

[181] Sibov T.T., Pavon L.F., Miyaki L.A., Mamani J.B., Nucci L.P., Alvarim L.T., Silveira P.H., Marti L.C., Gamarra L. (2014). Umbilical cord mesenchymal stem cells labeled with multimodal iron oxide nanoparticles with fluorescent and magnetic properties: Application for in vivo cell tracking. Int. J. Nanomed..

[182] Liu H., Tan Y., Xie L., Yang L., Zhao J., Bai J., Huang P., Zhan W., Wan Q., Zou C. (2016). Self-assembled dual-modality contrast agents for non-invasive stem cell tracking via near-infrared fluorescence and magnetic resonance imaging. J. Colloid. Interface Sci..

[183] Park J.S., Park W., Park S.J., Larson A.C., Kim D.H., Park K.H. (2017). Multimodal magnetic nanoclusters for gene delivery, directed migration, and tracking of stem cells. Adv. Funct. Mater..

[184] Wang L., Neoh K.-G., Kang E.-T., Shuter B., Wang S.-C. (2010). Biodegradable magnetic-fluorescent magnetite/poly (dl-lactic acid-co-α, β-malic acid) composite nanoparticles for stem cell labeling. Biomaterials.

[185] Xie X., Liu W., Zhu W., Zhang G., Dai Y., Wu J., Nie H., Lei L. (2022). A cell penetrating peptide-modified magnetic/fluorescent probe for in vivo tracking of mesenchymal stem cells. J. Biomed. Mater. Res. Part. A.

[186] Chen D., Wan D., Wang R., Liu Y., Sun K., Tao X., Qu Y., Dai K., Ai S., Tao K. (2018). Multimodal nanoprobe based on upconversion nanoparticles for monitoring implanted stem cells in bone defect of big animal. ACS Biomater. Sci. Eng..

[187] Tang Y., Zhang C., Wang J., Lin X., Zhang L., Yang Y., Wang Y., Zhang Z., Bulte J.W., Yang G.Y. (2015). MRI/SPECT/fluorescent tri-modal probe for evaluating the homing and therapeutic efficacy of transplanted mesenchymal stem cells in a rat ischemic stroke model. Adv. Funct. Mater..

